# Ritual’s collective effervescence, awe, and social identity: psychosocial effects of the Pasto carnival

**DOI:** 10.3389/fpsyg.2025.1566499

**Published:** 2025-11-11

**Authors:** Camilo Rincón-Unigarro, Silvia da Costa-Dutra, María-José Cisneros-Rodríguez, Laura Díaz-Fuentes, Natalia Giraldo-Castillo, Angélica Murcia-Infante, Darío Páez, Mariana Vargas-López, Santiago Vásquez-Velásquez

**Affiliations:** 1 Universidad de La Sabana, Chía, Colombia; 2 Universidad de Zaragoza, Teruel, Spain; 3 Facultad de ciencias sociales y Humanas de Teruel Universidad de Zaragoza, Zaragoza, Spain; 4 Centro de Investigacion, Innovacion Creación, Vicerrectoría de Investigación y Postgrado, Escuela de Medicina, Facultad de Ciencias de la Salud, Universidad Católica de Temuco, Temuco, Chile; 5 Universidad Andrés Bello, Santiago, Chile; 6 Programa doctorado Educacion y Ciencias Sociales, Facultad de Educacion y Ciencias Sociales, Universidad Andres Bello, Santiago, Chile

**Keywords:** superordinate identities, identification with all humanity (IWAH), intangible cultural heritage, self-transcendent emotions, collective action, collective gatherings, collective effervescence

## Abstract

**Introduction:**

This study analyzes the experience of participating in the 2023 *Carnaval de Negros y Blancos*, a festive event that emphasizes southwestern Colombia’s African, Andean, and European traditions.

**Methods:**

We surveyed a total of 163 participants, including dancers (*n* = 73, 44.79%), players (*n* = 47, 28.83%), and other artists (*n* = 43, 26.33%). Questions were asked about participation (type of role, number of days at the festival) and the quality of the experience according to the neo-Durkheimian model of collective gatherings (situated social identity, perceived shared attention and behavioral synchrony, fusion of personal and collective identity, perceived emotional synchrony, positive personal emotions, self-transcendent emotions, including awe, and experience of self-transcendence). The outcome variables were parochial altruism, identification with the community, the national, and humanity.

**Results:**

The participation experience was associated with all outcomes. The data supports that quality of participation in the event or collective effervescence, controlling for sociodemographic variables and intensity of participation, was associated to communal and national identification, but also to superordinate identification with all of humanity. Awe felt during *Carnaval de Negros y Blancos* correlates with the quality of experience, with social identification, and with superordinate identification with all of humanity. Mediation analyses show that collective effervescence influences community identity and parochial altruism through awe, but does not influence national or all of humanity’s identity.

**Discussion:**

We discuss why a local multicultural event partially reinforces superordinate identities, the limitations of the study, and our research approaches.

## Introduction

This article examines a festive collective gathering event and its potential effects on social identification. It seeks to address the question of whether an intangible cultural heritage event, such as a carnival, in addition to reinforcing local identities, reinforces supra-ordinate identities, in particular identification with all of humanity. Furthermore, it examines how the psychosocial processes that occur during them, in particular collective effervescence and the emotion of awe have effects on distinct levels of social identity.

*Carnaval de Negros y Blancos* (CNB) is an event that takes place annually from December 28 to January 6 in San Juan de Pasto, as an instance of cultural participation. It celebrates local identity and ingroup solidarity and can perhaps reinforce identification with all humanity (IWAH) (UNESCO, 2009). Despite previous evidence showing that the experience of collective gatherings increases different instances of superordinate identification (Rincón-Unigarro et al., 2024; Wlodarczyk et al., 2022), the role of highly localized events that celebrate ingroup identity on broader categories of identification is still unclear. Therefore, the study aims to fill this gap by linking social identification to the perceived collective effervescence at the gathering (da Costa et al., 2023; Rimé and Páez, 2023; Rincón-Unigarro et al., 2024), as well as to the transcendence emotion of awe felt during collective gatherings (Keltner, 2023).

There are multiple processes of social identification through which people can feel part of broader social categories. Among these are the superordinate ones, which include simultaneously identifying with multiple level groups (e.g., the representation of South Africa as the rainbow nation includes African, White, and Indian ethnic groups in the same category). These identities are associated with prosocial behavior toward members of ethnic in groups and outgroups (in this example, altruistic behavior toward all South Africans, be they black, white, or Indian) (Mason, 2023).

Three models can describe complex social identification processes in collective gatherings. According to the Common Group Identity model, collective gatherings reinforce the sense of belonging to an imagined community, transforming the representations of two opposing groups (e.g., “whites and blacks”) into a single, more inclusive one (e.g., nation, country). This perspective emphasizes the role of social re-categorization, whereby groups momentarily abandon subordinate group identities in favor of a superordinate category (Gaetner and Dovidio, 2012). However, groups are not always able to relinquish their identity. According to the Dual Categorization Model, collective gatherings reinforce dual identities, such as African Colombians or White Colombians (Dovidio et al., 1998). Lastly, according to the Mutual Intergroup Differentiation model, collective gatherings allow simultaneous awareness of subordinate and superordinate categories, allowing participants to maintain their respective social identities as the optimal condition for intergroup contact, so that the different groups within the nation groups of “black Colombians” and “white Colombians” are perceived as distinct but complementary (Dixon et al., 2018; Hogg, 2018).

Relevant to these models, collective encounters, gatherings, assemblies, and forms of collective behavior play a role in the formation, acquisition, and expansion of superordinate identities. During collective gatherings, the experience of collective effervescence involves group identification, behavioral and emotional synchronization processes (Bouchat et al., 2024). These include social identification processes like situated social identification (categorization as a member of the group participating in the collective gathering), and identity fusion (overlap of the personal self with the collective or group self). Also includes perceptive behavioral process (shared attentional focus and behavioral synchrony), as well as emotional ones (emotional synchrony, intense personal emotions, and self-transcendent emotions). Finally, collective gatherings include self-transcendence experiences (da Costa et al., 2023; Rimé and Páez, 2023). All these processes potentially lead to an experience of contact with self-transcendence social values and self-expansion, in which the self feels integrated into a larger or more transcendent collective (Rimé and Páez, 2023).

Within this type of collective behavior, evidence shows that intergroup contact experiences, particularly with larger collectives than the most local reference group, affect identification with all of humanity (IWAH) (Hamer and McFarland, 2023). IWAH refers to self-categorization and identification with people from all over the world, so that humanity is experienced as an in-group, independently of other, more specific, social categories (McFarland et al., 2019). Interestingly, there is supporting evidence on the effect of experiencing cosmopolitan events on superordinate identification. For instance, participating in an International Sports Tournament was related to an increase in IWAH (Wlodarczyk et al., 2022).

Therefore, there is a gap regarding the role of experiencing highly localized and culturally specific events on IWAH. Within this context, elements of intangible cultural heritage are a relevant case study, as they simultaneously recognize cultural authenticity and distinctiveness, while also embodying cosmopolitan values of a global shared humanity (Lázaro Ortiz and Jiménez De Madariaga, 2022).

Our main proposition is that, although CNB can reinforce dual identities or complementary identities, it can also generate a social recategorization process, not only nationally, but with all of humanity. This depends on the positive affective valence of the encounter, its carnivalesque character, and its emphasis on a long-standing ancestral cultural heritage (da Costa et al., 2023). In general, people identify more with the closest reference group, such as the community, city, or region; and, to a lesser extent, with the nation, and even less with superordinate groups, such as all of humanity (Wlodarczyk et al., 2022). IWAH implies conceiving of humanity as a global ingroup, and correlates with parochial altruism, concern for humanity, and knowledge of human rights. IWAH is also associated with the willingness to contribute to humanitarian aid, to grant intergroup forgiveness, and to have cross-cultural contact. Finally, IWAH is related to global social connectedness, like perceived closeness to other people, equal valuation of the lives of in-group and outgroup members, openness to experience, empathy, and universalistic values of tolerance (Grimalda et al., 2023; Hamer et al., 2017, 2019, 2021, 2022; McFarland et al., 2019; Newton and Ferenczi, 2024; Pizarro et al., 2021a).

Below, we will describe the Pasto CBN carnival to explain why it can give rise to superordinate identities and IWAH.

### CNB as an identitarian collective gathering

**Figure 1:**
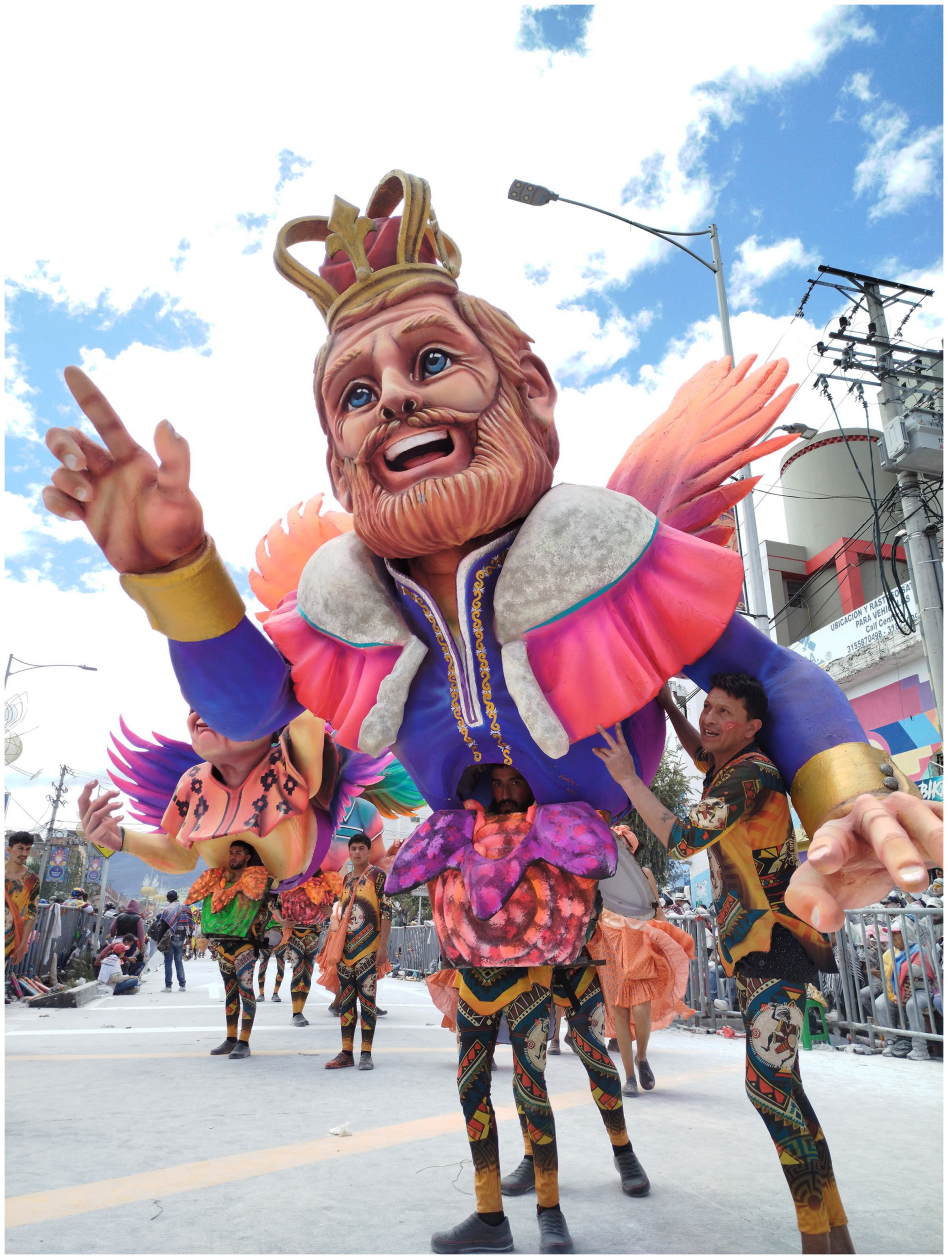
“Día de Asueto” by Guillermo Caicedo, a Parade Ensemble (Comparsa) on Día de Blancos (Day of Whites) 2025. This parade ensemble, composed of five sculptures, represents the association between the abolition of slavery and the colonial past of the region. Photograph by Camilo Rincón-Unigarro (2025).

CNB dates back to the early 19th century, linked to the quest for the abolition of slavery in South America (Muñoz, 2007). It has been formally organized in Pasto as a major festive event since 1927, continually evolving to preserve, incorporate, and develop diverse cultural expressions (Avendaño-Peña, 2024). These cultural expressions enact the collective memory of the city and its people, including its indigenous, colonial, republican, and contemporary periods (see Figure 1). Following its inclusion as intangible cultural heritage, CNB hosts consolidated collective action initiatives to safeguard its authenticity and sustainability (Rincón-Unigarro et al., 2024).

CNB expressions mostly take the form of massive collective gatherings, which are a potential source for the development of a superordinate social identity as they represent a syncretism of ingroup and outgroup characteristics. We analytically distinguish between three groups of participants at CNB according to their level of involvement in the event, as well as the connotation of its practice within each of the expressions of heritage in this setting.

**Figure 2:**
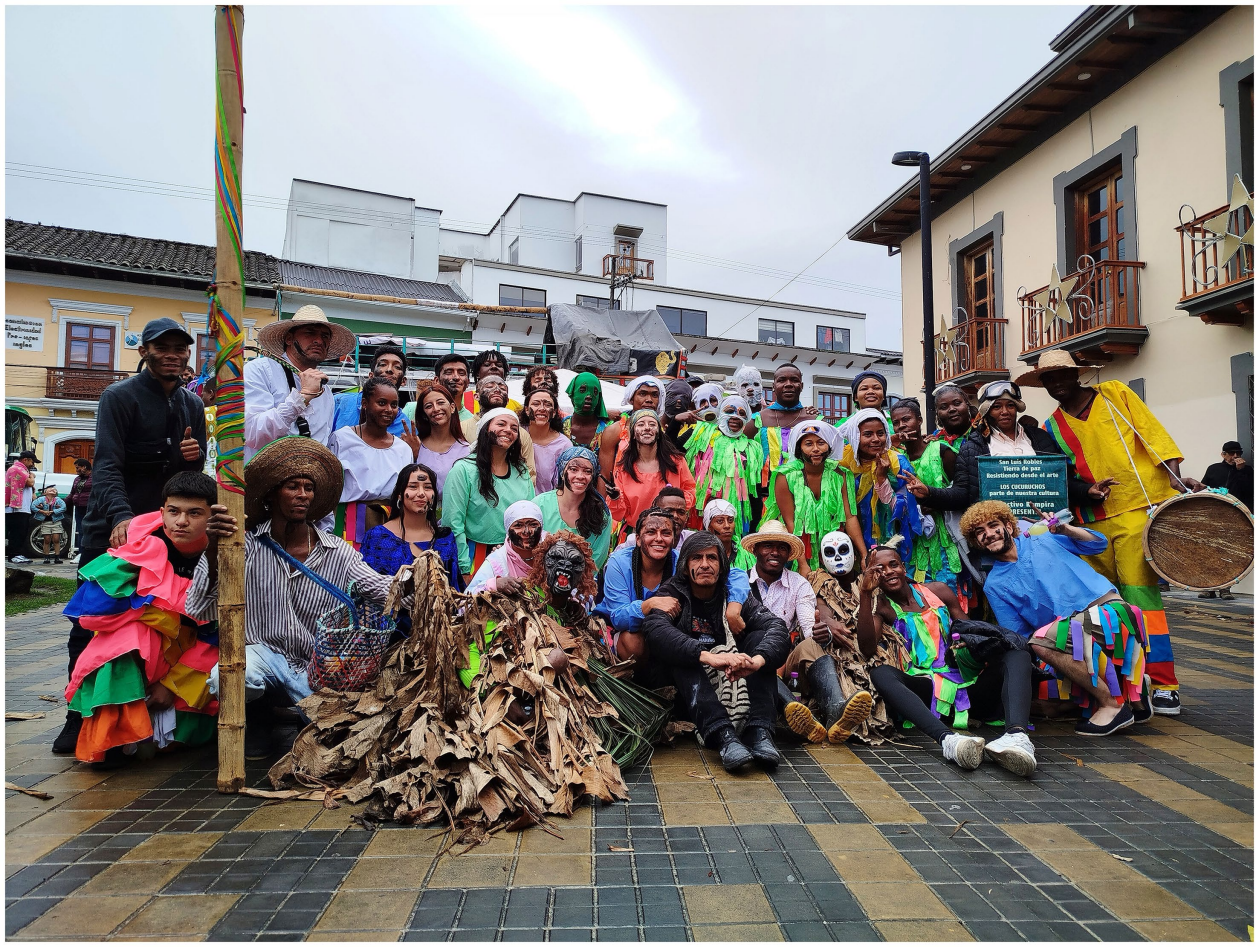
Members of Cucuruchos de la Fundación Rampira de San Luis Robles, Danza Pulso, Colectivo Raíz Afropacifico, and Fundación Cultural Vía Libre Pose after their performance “Los Cucuruchos” on Día de Negros (Day of Blacks) 2025. Both collectives lead resistance and community leadership processes for environmental sustainability and cultural representation. Photograph by Camilo Rincón-Unigarro (2025).

The first group involves participants taking part in the traditional practice of *Play*, who participate throughout all carnival days, but whose commemorative event is the Day of Blacks, celebrated on January 5th. In this ritual, which is the original expression of CNB, participants paint others’ faces with colored cosmetics in a narrative of interethnic contact that questions traditional social hierarchies (Sansón Rosas and Fusté-Forné, 2018). All participants of CNB, who take part in any of its main activities, are involved as *players*. Some cultural organizations dedicate events to the practice and narrative of play (see Figure 2).

**Figure 3:**
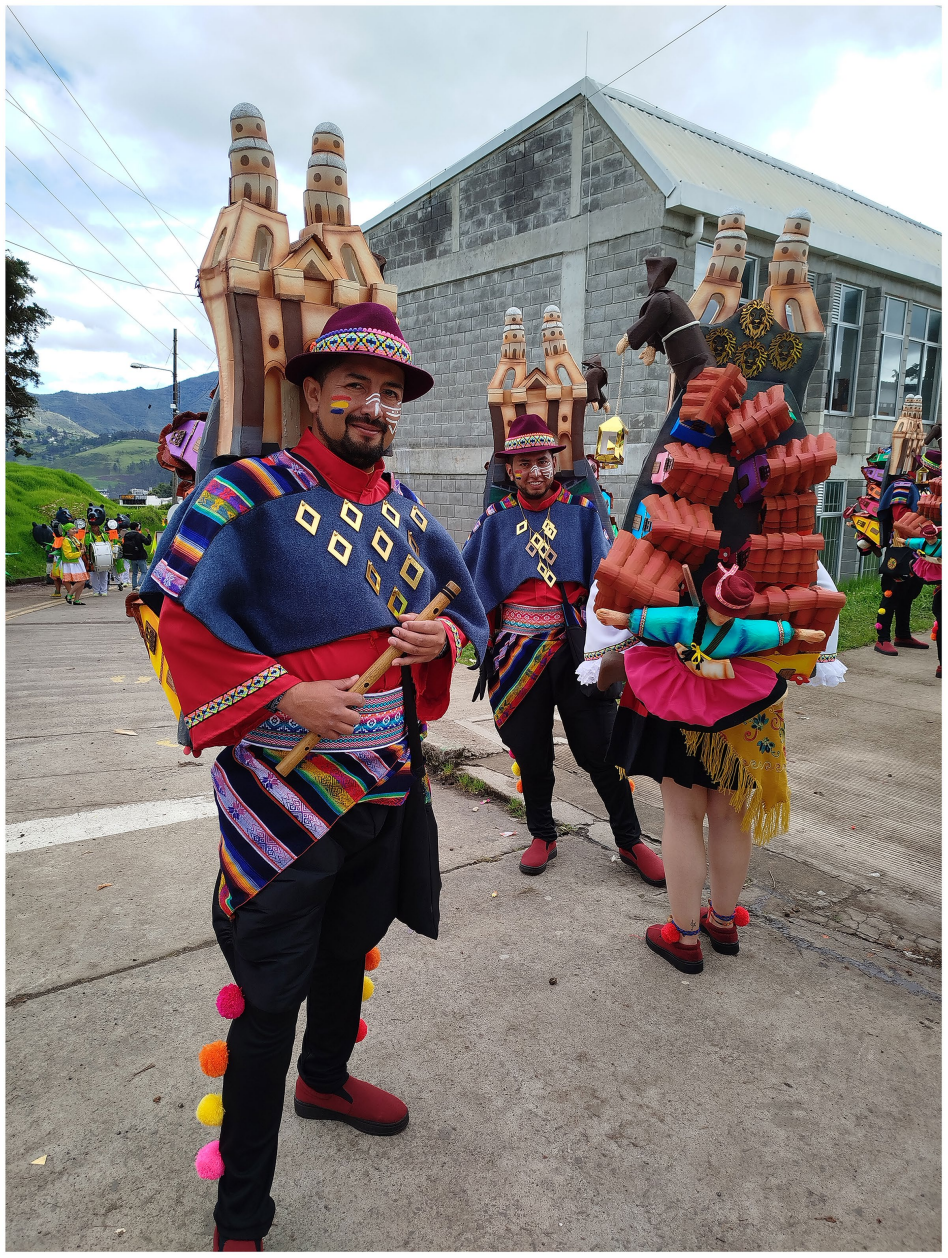
Members of Kinde Sicuris, a Street Band (Murga), show their attire before performing on Día de Blancos (Day of Whites) 2025. The attire represents symbols and elements of “El Colorado,” a historical and emblematic street in Pasto. Photograph by Camilo Rincón-Unigarro (2025).

**Figure 4:**
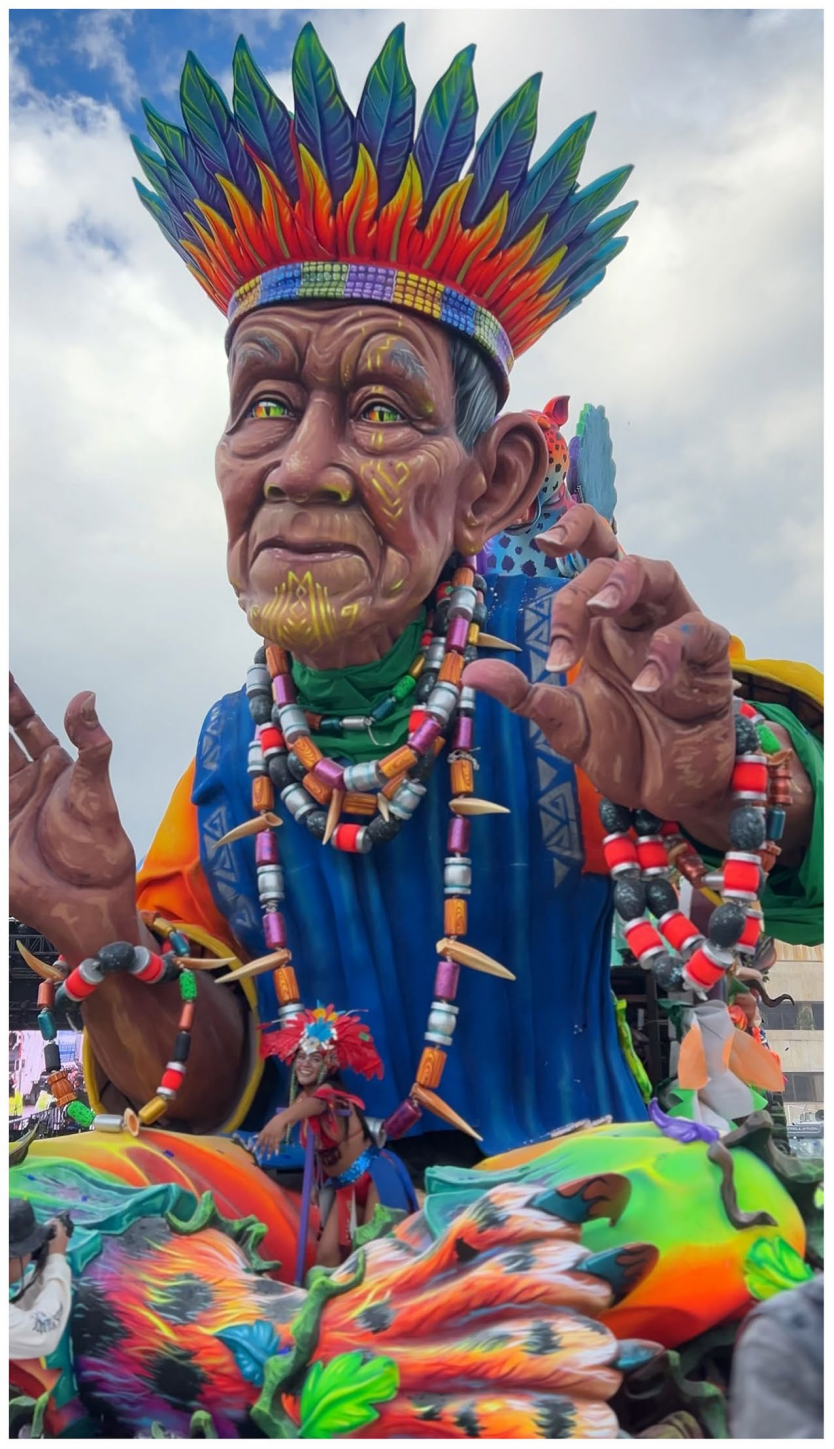
“La Vorágine,” by Leonard Augusto Zarama, an allegorical float (Carroza) represents the amazon rainforest, the connection between humans and nature, and indigenous resistance in South America. The float honors José Eustacio Rivera’s literary work and Taita Querubín Queta Alvarado’s resistance work as an Indigenous leader. Photograph by Laura María Pazmiño, 2025 (Instagram account: @pazmi1).

The second group of participants includes artists in all the main modalities, those who participate in the *Day of the Whites*, celebrated on January 6th. Celebrated since the early 20th century, the event evolved to incorporate and develop a set of artistic expressions that include the main modalities of CNB: costume, parade ensemble (comparsa), street band (murga), and allegorical float (carroza) (Muñoz, 2007). Each modality connotes and denotes social identities at multiple levels; some represent the local categories (see Figure 3); while others represent superordinate, identities (e.g., regions, animals, spirits, natural phenomena, and objects) (see Figure 4).

**Figure 5:**
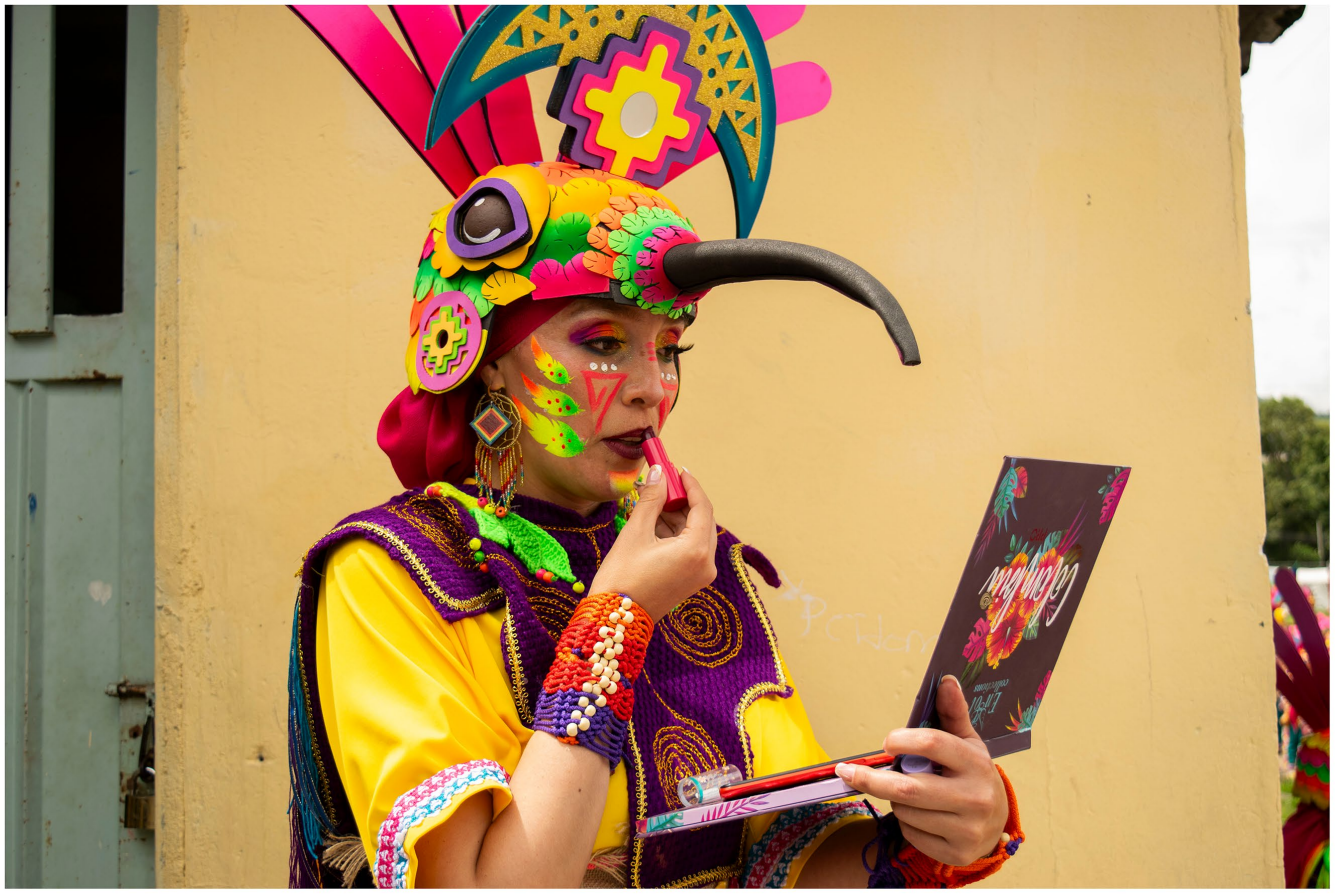
A Member of Danzantes del Cerrillo Choreographic Collective Prepares Before Their Performance “Buen Vivir, Sentir del Sur” at Canto a la Tierra 2025. The attire represents a hummingbird ornamented with a “Cruz del Sur” motif, symbols of the Andean region. Photograph by Laura María Pazmiño, 2025 (Instagram account: @pazmi1).

The third group of participants we considered are Dancers, those who participate as part of Choreographic Collectives on *Canto a la Tierra*, celebrated on January 3rd. This is one of the most recent events within CNB, held annually since the 1990s, and connects artists and performers with the symbology and folklore of Andean culture (Avendaño-Peña, 2024). *Canto a la Tierra* consists of the performance of thousands of dancers in the streets of the city with costumes and choreographies that represent the popular culture shared with other countries of the region (Argentina, Bolivia, Chile, Colombia, Ecuador, Peru, and Venezuela) (see Figure 4). The adaptation of foreign artistic elements within a highly localized patrimonial cultural expression allows a transversal cultural dialogue, but also motivates concern for patrimonial authenticity and local representation (Villanueva, 2021) (see Figure 5).

### Study objectives and hypotheses

The objective of this study is to contrast the relationship between the intensity of participation in a collective ritual (Pasto carnival CBN), the quality of the experience or collective effervescence, including the processes of social identification and identity fusion with the participants, with social integration, in particular communal identity, national identity, and identity with all of humanity. Specifically, we examine the role of the emotion of awe linked to collective ritual in social identification.

To advance knowledge on how collective encounters reinforce social identities, particularly supra-ordinate ones, first, we draw upon research on social identity, a form of self-categorization that arises from belonging to a group. People’s self-concept and self-esteem are strongly influenced when people categorize themselves as members of a group and strongly identify with it (Hornsey, 2008). High social identification is associated with favoritism toward the in-group in a meta-analysis. In turn, favoritism toward the in-group is related to high personal self-esteem. However, in two meta-analyses high personal self-esteem is related to a positive evaluation of out groups, suggesting that self-categorization, in-group favoritism and strong self-esteem coexist with a more benign view of out groups (Rivera et al., 2024).

Previous studies have shown that active participation in collective gatherings, in comparison with non-participants or audience, are related to higher situated social identity or identification with co-participants and with large in-groups (with demonstrators and the feminist social movement, for example, Zumeta et al., 2020). Active participation was also related to higher collective effervescence, behavioral and emotional synchrony, and personal wellbeing, including self-esteem (Pizarro et al., 2022; Zumeta et al., 2020; Xygalatas, 2022).

*H1*: Participants involved in more active groups (artistic modalities vs. players) and more intensive forms of participation (more days and more modalities) will report higher levels of the socio-cognitive (social identity); behavioral and emotional processes of collective effervescence gatherings.

Second, we base our study on research on collective gatherings showing that the degree of participation in collective rituals is associated with social integration and social identification (Pizarro et al., 2022; Xygalatas, 2022). Research on intangible cultural heritage and social identities shows that taking part in these highly localized identity expressions increases community identification, inclusion, cohesion, and resilience with reference groups (Chng and Narayama, 2017; Hebert et al., 2012; Lázaro Ortiz and Jiménez De Madariaga, 2022; Zabala et al., 2023). Moreover, participation in heritage collective gatherings also predicts the formation of more superordinate identities, such as the ethnic, national, and religious levels (Xygalatas, 2022).

In this regard, Hopkins et al. (2016) found that, during the Hindu Magh Mela festival in India, those who participated with greater involvement in the event felt more identified with members of the crowd, at the same time, perceiving themselves as members of the collective of Hindu believers. Khan et al. (2016) stated that Hindus participating in a Mauritian religious ritual celebrated as a national holiday, while experiencing greater situated social identity, also reported greater national identification. Not only did they perceive Hindus as more Mauritian, but they also saw Christians and Muslims as more Mauritian. Therefore, these studies show that collective gatherings, despite being highly localized expressions of culture, can also promote broader levels of social identification, as well as increase self-transcendence emotions and social cohesion. Participation in collective gatherings will be associated particularly to awe, a self-transcendence emotion which leads self to expand into greater realities, increase integration of personal self with larger entities, promotes prosocial behavior, evokes greater social connectedness and heightened sense of meaning, connecting the individual to a larger narrative, ideology, or purpose (Keltner, 2023). Finally, studies show that participants in collective gatherings, in comparison with audience or no participants report high positive affect, social identification and transcendence beliefs and values (Páez et al., 2015).

*H2*: Participants involved in more active groups (artistic modalities vs. players) and more intensive forms of participation (more days and more modalities) will report higher levels of awe (H2a), parochial altruism (H2b), community identification (H2c), national identification (H2d), IWAH bond (H2e), and IWAH concern (H2f).

Third, shared collective experiences, including collective effervescence, during rituals reinforce identity fusion. Identity fusion is a process in which the personal and social self overlap (Gómez et al., 2020). Shared experiences increases identity fusion with the immediate reference group (e.g., feminist demonstrators or a battalion of fighters); but also with broader categories, such as the nation, political, or religious movements (e.g., feminist social movement, the community of Muslim believers) (Lobato and Sainz, 2019; Pizarro et al., 2022). Meta-analytic evidence confirms that identity fusion is a stronger predictor of pro-group behavior than social identification, although affectively loaded social identity scales have the same predictive power (Varmann et al., 2024). We expect that identity fusion, as well as situated social identity, will be associated with collective gatherings outcome (Bouchat et al., 2024). A meta-analysis confirms that quality of collective gatherings experience, particularly situated social identification and perceived emotional synchrony (Bouchat et al., 2024), together with identity fusion, behavioral synchrony, shared attention, emotional synchrony, self-transcendence emotions and experiences, were associated to social identification with large groups (Pizarro et al., 2022).

*H3*: The sociocognitive, behavioral and emotional processes of collective gatherings, like identity fusion, will be consistently associated with awe (H3a), parochial altruism (H3b), community identification (H3c), national identification (H3d), IWAH bond (H3e), and IWAH concern (H3f).

Fourth, the experience of collective effervescence in collective gatherings can lead to self-transcendence (e.g., feeling connected to something larger than oneself). A meta-analysis supports that collective effervescence, assessed as perceived emotional synchrony during collective gatherings, is associated with self-transcendent emotions, beliefs, and values (e.g., universalism and justice) (Pizarro et al., 2022; Schwartz, 2021). Self-transcendence emotions are distinctive in that those who experience them focus outward toward others, rather than toward themselves (Bouchat et al., 2024; Cusi et al., 2022; Pizarro et al., 2022). Among these, awe is a key proximal outcome of appraising the collective encounter as larger than oneself, creating a sense of immense power and unity (Keltner, 2023). Empirical evidence shows that these emotions shift the focus from self-absorption to concern for others, environmental issues, group interests, and IWAH (Pizarro et al., 2021a, 2021b). Hence, these studies show that participation in a collective encounter, even when culturally localized, could promote self-transcendence, predicting experiences of self-transcendent emotions, altruism, or IWAH.

*H4*: The self-transcendent emotion of awe mediates the effect of participating in CNB on parochial altruism (H4a), community identification (H4b), national identification (H4c), IWAH bond (H4d), and IWAH concern (H4e).

Our research contributes to the understanding of how intangible cultural heritage is related to self-transcendent emotions, parochial altruism, and social identification. Cultural heritage is deeply rooted in the social beliefs about the group’s past and future and it can reinforce the group’s collective identity through activities carried out in collective gatherings (Alegría Licuime et al., 2018; Cotte et al., 2024). Furthermore, empirical research shows that in countries marked by violent conflict and social inequality, safeguarding cultural heritage promotes peacebuilding. Enhancing cultural heritage allows its members to recognize and signify the shared experience as a group, strengthening their social fabric, while providing a legitimate mechanism to create a future orientation for the community (Carter and Guerra, 2022; Kolk, 2020; Lixinski and Williams, 2024; Munawar and Symonds, 2022). These studies, however, focus on the effect of expressions of in-group culture on proximal identities, but not on the superordinate level of identification. Following these empirical studies, the original contribution of this study is to assess how highly localized events, such as the CNB, work as a source of superordinate identities that promote intergroup cooperation and the reaffirmation of common goals (Reimer et al., 2017; Uluğ and Cohrs, 2017; Wulf, 2022).

## Method

### Procedure

Data collection took place in August 2023 during a fieldwork visit to Pasto, Colombia. Artistic collectives were already creating their performances for CNB 2024. Participants received the invitation to participate in a study about social identities in Pasto and the impact of CNB on its participants. Questions were framed to ask for participation experiences during the 2023 CNB, 7 months before data collection.

We used a snowball sampling technique to recruit participants involved in the 2023 CNB by asking leaders of artistic collectives to complete the survey and then share it with other artists who participated in their group. We chose this method due to the informal organization of artistic groups and the absence of a centralized list of participants, which made it difficult to conduct a probabilistic sampling. Snowball sampling is a well-supported technique in social science research, especially for reaching hidden or network-based populations within cultural and community contexts (TenHouten, 2017; Kirchherr and Charles, 2018; Raifman et al., 2022). While this method may introduce sampling biases such as network homogeneity or overrepresentation of socially connected individuals (Došek, 2021), we mitigated these risks by initiating recruitment from diverse collectives and participation modalities to increase sample heterogeneity.

### Participants

Sample composition in terms of sex, socioeconomic status, race, civil status, occupation, education level, events attended, and modalities of participation are reported in Table 1. Modality of participation distinguished those who participated in *choreographic collectives*; *other artists*, involving any of the other forms of artistic performance, excluding choreographic collectives; and *players,* involving participants who took part in the event outside any form of artistic modality.

**Table 1:** Sample composition.

Variable	Category	*n*	%
Sex	Male	85	52.15
	Female	77	47.24
	Other	1	0.61
Socioeconomic Status	1	31	19.02
	2	65	39.88
	3	30	18.41
	4	22	13.50
	5	7	4.29
	6	7	4.29
Race	Black	2	1.23
	Other	6	3.68
	Mulata	7	4.29
	Indigenous	17	10.43
	White	25	15.34
	Mestiza	104	63.80
Civil status	Other	5	3.07
	Divorced	6	3.68
	Married	18	11.04
	Lives with Partner	27	16.56
	Single	106	65.03
Occupation	Retiree	2	1.23
	Neither Working, Nor Looking for a Job	2	1.23
	Housekeeping	4	2.45
	Looking for a Job	23	14.11
	Working	55	33.74
	Student	76	46.63
Education	Other	3	1.84
	Primary	1	0.61
	Secondary	62	38.04
	Non-University Higher Education	10	6.14
	University	70	42.95
	Postgraduate	16	9.82
Modality	Choreographic Collective	73	44.79
	Play	70	42.94
	*Arcoíris en el Asfalto*	57	34.97
	Concert	52	31.90
	Street Band (*Murga*)	35	21.47
	Attendant	31	19.02
	Other	25	15.34
	Parade Ensemble (*Comparsa*)	22	13.50
	*Años Viejos* Parade	22	13.50
	Float (*Carroza*)	19	11.66
	Classic Car Parade	13	7.98
	Individual Costume	5	3.07
	Organization and Logistics	4	2.45
	Press	1	0.61
Group	Choreographic Collectives	73	44.79
	Players	47	28.83
	Other Artist	43	26.38

Street band, parade ensemble, años viejos parade, float, individual costume formed the group Other Artists. Play, Arcoíris en el Asfalto, Concert, Attendant, Other, classic car parade, organization and logistics, and press formed the group Players.

### Measures

#### Involvement in the event

##### Number of days of participation (*ad hoc*)

Participants reported how many *days* they participated in at CNB. Even when some modalities prepare and train for the main event all year long, we exclusively consider the pre-carnival and carnival periods, from December 28 to January 8. Respondents indicated which days they had taken part in, and then we calculated the sum of days of participation.

##### Number of modalities of participation (*ad hoc*)

Participants reported the number of *modalities* that they participated in at CNB (see Table 1). Modalities were assessed through a checklist of the 15 central expressions associated with Carnival (see Appendix B). Respondents indicated which of these activities they had taken part in, and then we calculated the sum of modalities of participation.

##### Group of participation (*ad hoc*)

We created a categorical variable to distinguish between three broad groups of participation at CNB, according to the modality of participation. If the respondent participated in a choreographic collective, it was assigned to the *Dancer* group, independently of its involvement in any other modality. If the respondent did not participate in a choreographic collective but participated in the other artistic modalities (street band, parade ensemble, *Años Viejos* parade, floats, or individual costume), it was assigned to the *Other Artist* group. If the respondent did not participate in any of the artistic modalities, but participated in play, *Arcoíris en el Asfalto*, concerts, attendants, classic car parade, organization and logistics, press, and other participation, it was assigned to the *Player* group.

#### Processes of collective gatherings (neo Durkheimian model)

##### Situated social identification

Three items adapted (Novelli et al., 2013; see da Costa et al., 2023) assessed participants’ identification with others at CNB. Response options ranged from 1 = “Strongly Disagree” to 7 = “Strongly Agree.” E.g., “I felt strong bonds with the other people who were in the collective.” Cronbach’s *α* = 0.85.

##### Perceived shared attention

Three items (Zumeta et al., 2020; see enlarged scale at da Costa et al., 2023), measured shared attention. E.g., “The people who participated in the collective gathering simultaneously concentrated or focused their attention on the same.” Response options ranged from 1 = “Not at all” to 7 = “Very much.” Cronbach’s *α* = 0.86.

##### Perceived behavioral synchrony

Four items (Zumeta et al., 2020; see enlarged scale at da Costa et al., 2023) were used that measured behavioral synchrony. E.g., “The people who participated in the collective gathering were acting in harmony.” Response options ranged from 1 = “Not at all” to 7 = “Very much.” Cronbach’s *α* = 0.88.

##### Identity fusion scale

Six items (Gómez et al., 2011; view reduced version at da Costa et al., 2023) measured a process in which the personal self and the social self-become highly aligned and emotionally integrated. Unlike social identity, identity fusion implies that individuals maintain a strong sense of individuality, but place it at the service of the group, which can lead to extreme behaviors on behalf of the group, including self-sacrifice, due to a perceived visceral unity with other group members (Gómez et al., 2020; Swann et al., 2012). E.g., “I wanted to do more for my group than any other group member would want to do.” Response options ranged from 1 = “Strongly Disagree” to 7 = “Strongly Agree.” Cronbach’s *α* = 0.76.

##### Perceived emotional synchrony (PES)

Six items derived from the original scale Wlodarczyk et al. (2020) measured perceived emotional synchrony (sense of union and intense sharing) with co-participants during CNB. E.g., “We felt that we were a whole.” Response options ranged from 1 = “Not at all” to 7 = “Very Much.” Cronbach’s *α* = 0.91.

##### Perceived positive personal emotions

Three items (Novelli et al., 2013; adapted by Wlodarczyk et al., 2020) measured the positive emotions of transcendence felt at CNB. E.g., “I felt fulfilled.” Response options ranged from 1 = “Not at all” to 7 = “Totally.” Cronbach’s *α* = 0.82.

##### Perceived transcendent personal emotions

Six items (based on Fredrickson, 2009, adapted by Wlodarczyk et al., 2020 and da Costa et al., 2023) measured the positive emotions of transcendence felt at CNB. E.g., “I felt love, closeness, trust.” Response options ranged from 1 = “Not at all” to 7 = “Totally.” Cronbach’s α = 0.95.

##### Perceived negative personal emotions

Three items measured the negative emotions felt at CNB (based on Fredrickson, 2009, adapted by Wlodarczyk et al., 2020). E.g., “I felt stressed, nervous, or overwhelmed.” Response options ranged from 1 = “Not at all” to 7 = “Totally.” Cronbach’s *α* = 0.91.

##### Transcendent experience

Four items (Gabriel et al., 2020, adapted by Wlodarczyk et al., 2020 and da Costa et al., 2023) measured the degree of transcendence experienced by people during CNB. E.g., “I felt that there was something associated with values and ideals in the event.” Response options ranged from 1 = “Strongly Disagree” to 7 = “Strongly Agree.” Cronbach’s α = 0.83.

#### Outcomes of collective gatherings

##### Self-transcendent emotion of awe

Seven items derived from the original scale (Pizarro et al., 2018; see short version by da Costa et al., 2023) measured the experience of awe during CNB. E.g., “I felt in the presence of something great.” Response options ranged from 1 = “Not at all” to 7 = “Very much.” Cronbach’s α = 0.84.

##### Parochial altruism

Five items scale (Zumeta et al., 2020; adapted by da Costa et al., 2023) assessed the behavioral intention of participants to collaborate in actions, organizations, and initiatives in favor of the rights of the group to which the participants identified with the most: Colombia (nation), Nariño (department), or Pasto (city). E.g., “I am willing to commit 2 h a week to collaborate with an association that organizes mobilizations.” Response options ranged from 1 = “Not at all” to 5 = “A lot.” Cronbach’s α = 0.86.

##### Community identification

Identification with All Humanity Scale (IWAH) assessed the degree to which participants felt *close*, had *in common,* were *concerned*, and were *willing to help* their own community (McFarland et al., 2012; adapted by Wlodarczyk et al., 2022). E.g., “How close do you feel to people in your community”? Response options ranged from 1 to 5, Cronbach’s α = 0.86.

##### National identification

Identification with all humanity scale (IWAH), assessed the degree to which participants felt *close*, had *in common, were concerned*, and were *willing to help* their nation (McFarland et al., 2012; adapted by Wlodarczyk et al., 2022). E.g., “How much do you identify with (that is, feel a part of, feel love toward, have concern for) Colombians?” Response options ranged from 1 to 5, Cronbach’s α = 0.85.

##### Supraordinate or humankind identity

*IWAH Bond.* Two items Identification with All Humanity Scale (IWAH) assessed the degree to which participants felt *close* and had *in common with* all of humanity (McFarland et al., 2012; adapted by Wlodarczyk et al., 2022). E.g., “How much would you say you have in common with people all over the world? Response options ranged from 1 to 5. *IWAH Concern.* Identification With All Humanity Scale (IWAH) assessed the degree to which participants felt *concerned* and *willing to help* all of humanity. E.g., “How much would you say you care (feel upset, want to help) when bad things happen to people anywhere in the world.” Response options ranged from 1 to 5. Global reliability for four items of Identification with all Humanity sub scale was satisfactory, Cronbach’s α = 0.84.

##### Control variables

Items were included to assess age, sex, socioeconomic level (Colombia’s population is stratified by the Government in six socioeconomic strata, from 1 to 6), ideological orientation (response scale 1 = left to 7 = right), and importance attributed to religion (response scale 1 = not at all to 4 = very much).

### Data analysis

We conducted ANOVA single-factor analysis and Pearson’s correlation analyses to test for differences in the dependent variables across modalities of participation (H1 and H2). To test H3, we conducted a series of correlations and multiple linear regressions of collective effervescence, along with a series of control variables, as predictors of dependent variables. For these models, no outliers were excluded (see Appendix B). Finally, to test H4, we conducted a series of mediation analyses with 5,000 bootstraps, using awe as a mediator between collective effervescence and the outcome variables.

To test our hypotheses related to collective effervescence, we created a new variable called Total Effervescence, which is an average score of all of the variables within the neo-Durkheimian model, except for negative personal emotions. We excluded negative personal emotions from Total Effervescence, given the festive connotation of CNB, and considering that negative personal emotions were negatively correlated with the neo-Durkheimian model as reported by Rincón-Unigarro et al. (2024).

## Results

Table 2 presents descriptive statistics for the variables in the study. Multiple forms of participation were common in the sample, as, on average, participants took part at CNB for 3.39 days and in 2.70 modalities. Concerning the processes of collective gatherings, except for negative emotions. All mean scores were above the midpoint of the scales. Concerning the hypothesized outcomes of participation at collective gatherings, the mean scores of awe, parochial altruism, community identification, national identification, IWAH bond, and IWAH concern were slightly above the midpoint of their corresponding scales.

**Table 2:** Descriptive statistics of study variables.

Variable	*n*	*M*	SD	Min.	Max.
Awe	162	5.60	1.14	1.50	7
Parochial altruism	162	4.31	0.86	1.00	5
Community identification	162	4.24	0.80	1.25	5
National identification	162	3.99	0.86	1.25	5
IWAH bond	162	3.10	1.10	1.00	5
IWAH concern	162	3.72	1.07	1.00	5
# Days of participation	163	3.39	2.24	1.00	10
# Modalities of participation	163	2.70	1.91	1.00	10
Situated social identification	163	5.92	1.09	3.00	7
Shared attention	163	5.53	1.12	2.00	7
Behavioral synchrony	163	5.70	1.05	2.75	7
Emotional synchrony	163	5.61	1.18	1.83	7
Identity fusion	163	5.21	1.15	2.00	7
Positive emotions	163	6.28	0.90	3.00	7
Transcendent emotions	163	6.35	0.93	2.83	7
Negative emotions	163	2.65	1.86	1.00	7
Transcendent experience	162	6.22	0.90	2.75	7
Total effervescence	162	5.85	0.78	3.38	7
Ideological orientation	162	3.69	1.79	1.00	7
Relevance attributed to religion	162	2.90	1.06	1.00	4
Age	163	28.66	11.84	18	66
Socioeconomic status	162	2.57	1.30	1	6

### Preliminary and descriptive analyses

Given that participants responded to dependent and independent variables at a single time point, we performed Harman’s single-factor test for common method bias with all items entered simultaneously. We used exploratory factor analysis (EFA), with a fixed one-factor solution, using an analysis based on a correlation matrix for Harman’s test. The unrotated factor solution revealed that a single factor accounted for 32.1% of the total variance, which is below the 50% threshold commonly used to indicate substantial common method variance. These results suggest that common method bias is not a concern in the present study.

We found two statistically significant differences between control variables across groups. Concerning ideological orientation, dancers (*M* = 3.27, *SD* = 1.78) were significantly more oriented toward the ideological left than players (*M* = 4.21, *SD* = 1.69), *F*(2, 159) = 4.27, *p* = 0.016, η_p_^2^ = 0.05. In addition, concerning age, dancers (*M* = 27.66, *SD* = 11.36) and players (*M* = 25.13, SD = 7.30) were significantly younger than other artists (*M* = 34.23, *SD* = 14.60), *F* (2, 160) = 7.71, *p* = 0.001, η_p_^2^ = 0.09. There were no other statistically significant differences across control variables.

### Participation at CNB and its outcomes

To test H1, we conducted a series of ANOVAs comparing three modalities of participation at CNB (see Table 3). Partially supporting H1, ANOVA results comparing participation groups show statistically significant differences in the following processes of the neo-Durkheimian model: positive personal emotions (η_p_^2^ = 0.139), personal transcendent emotions (η_p_^2^ = 0.131), transcendent experience scores (η_p_^2^ = 0.060), and total effervescence (η_p_^2^ = 0.054). However, no other mean differences were statistically significant between participation groups.

**Table 3:** Means, standard deviations (in parentheses), and ANOVAs between groups.

Variable	Groups			*F*
	Choreographic collectives (*n* = 73)	Other artists (*n* = 42)	Player (*n* = 47)	
Awe	5.88 (0.93)	5.67 (1.12)	5.12 (1.31)	6.92**
Parochial altruism	4.58 (0.64)	4.33 (0.76)	3.87 (1.07)	10.95***
Community identification	4.26 (0.78)	4.55 (0.56)	3.92 (0.92)	7.50***
National identification	3.96 (0.88)	4.23 (0.73)	3.80 (0.90)	2.76
IWAH bond	3.03 (1.14)	3.30 (1.14)	3.05 (1.02)	0.87
IWAH concern	3.57 (1.12)	4.11 (0.83)	3.61 (1.12)	3.86*
Situated social identity	6.06 (1.05)	5.89 (1.17)	5.70 (1.07)	1.58
Shared attention	5.50 (1.22)	5.58 (1.06)	5.52 (1.04)	0.07
Behavioral synchrony	5.76 (1.07)	5.69 (1.02)	5.63 (1.08)	0.23
Identity fusion	5.22 (1.21)	5.42 (0.91)	4.99 (1.25)	1.58
Emotional synchrony	5.75 (1.11)	5.79 (1.05)	5.23 (1.32)	3.52*
Positive emotions	6.56 (0.66)	6.36 (0.82)	5.77 (1.09)	12.92***
Transcendent emotions	6.66 (0.63)	6.39 (0.73)	5.86 (1.25)	12.05***
Negative emotions	2.68 (1.94)	2.38 (1.65)	2.87 (1.92)	0.79
Transcendent experience	6.41 (0.72)	6.26 (0.80)	5.89 (1.12)	5.11**
Total effervescence	5.99 (0.70)	5.91 (0.69)	5.57 (0.90)	4.50*

*p < 0.05; **p < 0.01; ***p < 0.001.

*Post hoc* analyses using the Bonferroni test show that Players reported significantly lower scores of personal positive emotions than both Choreographic Collectives (Cohen’s *d* = 0.94, *p* < 0.001) and Other Artists (Cohen’s *d* = 0.71, *p* = 0.003). The same result was found with transcendent emotions, with higher scores among players than Choreographic Collectives (Cohen’s *d* = 0.92, *p* < 0.001) and Other Artists (Cohen’s *d* = 0.61, *p* = 0.013). Complementarily, Players reported significantly lower scores of transcendent experience than Choreographic Collectives (Cohen’s *d* = 0.60, *p* = 0.005), but not with other artists (Cohen’s *d* = 0.42, *p* = 0.147). The same occurred with Total Effervescence with the Choreographic Collectives (Cohen’s *d* = 0.012, *p* = 0.012), but not with Other Artists (Cohen’s *d* = 0.45, *p* = 0.110).

We also analyzed a correlation matrix between the variables in the study (see Table 4). Partially supporting H1, results show a statistically significant and positive correlation between the number of days and modalities of participation at CNB with total collective effervescence, *r* = 0.19 and *r* = 0.15. Involvement correlates specifically with positive personal emotions, transcendent personal emotions, and transcendent experience.

**Table 4:** Pearson’s Correlation Matrix of awe, parochial altruism, social identification, intensity and quality of the participation in CBN.

Variable	1	2	3	4	5	6	7	8	9	10	11	12	13	14	15	16	17	18	19	20	21
1. Awe																					
2. Parochial altruism	0.56***	—																			
3. Community identification	0.41***	0.57***	—	0.00																	
4. National identification	0.28***	0.38***	0.70***	—																	
5. IWAH bond	0.17*	0.24**	0.36***	0.64***																	
6. IWAH concern	0.21**	0.32***	0.54***	0.71***	0.60***																
7. # of days	0.19*	0.23**	0.19*	0.19*	0.10	0.13															
8. # of modalities	0.16*	0.23**	0.08	0.08	0.01	0.07	0.59***														
9. Situated social identity	0.38***	0.38***	0.34***	0.24**	0.14	0.18*	0.19*	0.11													
10. Shared attention	0.22**	0.26***	0.24**	0.11	0.12	0.03	0.14	0.09	0.49***												
11. Behavioral synchrony	0.32***	0.24**	0.31***	0.32***	0.18*	0.22**	0.14	0.12	0.45***	0.57***											
12. Identity fusion	0.30***	0.21**	0.33***	0.29***	0.19*	0.14	0.02	−0.05	0.38***	0.38***	0.27***										
13. Emotional synchrony	0.45***	0.37***	0.48***	0.35***	0.21**	0.25**	0.08	0.08	0.57***	0.47***	0.52***	0.52***									
14. Personal positive emotions	0.61***	0.61***	0.46***	0.31***	0.14	0.19*	0.24**	0.21**	0.54***	0.44***	0.40***	0.39***	0.57***								
15. Personal transcendent emotions	0.61***	0.61***	0.51***	0.31***	0.14	0.18*	0.23**	0.18*	0.53***	0.45***	0.42***	0.41***	0.62***	0.86***							
16. Personal negative emotions	0.04	−0.12	−0.28***	−0.06	0.01	−0.04	−0.12	−0.06	−0.02	−0.09	−0.08	−0.02	−0.09	−0.13	−0.07						
17. Transcendent experience	0.44***	0.56***	0.44***	0.30***	0.09	0.19*	0.20*	0.17*	0.51***	0.51***	0.43***	0.41***	0.46***	0.66***	0.72***	−0.04					
18. Total effervescence	0.55***	0.53***	0.51***	0.37***	0.21**	0.23**	0.19*	0.15*	0.75***	0.73***	0.70***	0.64***	0.80***	0.79***	0.82***	−0.09	0.77***				
19. Socioeconomic status	0.02	−0.08	0.03	0.09	0.19*	0.09	0.22**	0.06	−0.01	0.09	0.07	0.15	0.13	0.00	−0.06	0.02	−0.13	0.05			
20. Ideological orientation	0.02	−0.09	0.02	0.08	0.10	0.06	−0.03	−0.13	0.07	0.14	0.33***	0.08	0.12	0.01	0.05	0.02	0.00	0.14	0.31***		
21. Religion relevance	0.05	0.01	0.04	0.09	0.11	−0.02	−0.15	−0.06	0.18*	−0.01	0.13	0.19*	0.13	0.08	0.08	0.08	0.07	0.15	0.05	0.23**	
22. Sex (Male)	−0.07	−0.09	−0.23**	−0.13	0.02	−0.11	−0.14	−0.01	−0.13	−0.02	−0.06	−0.03	−0.10	−0.08	−0.19*	0.07	−0.19*	−0.13	0.03	−0.06	−0.10

*p < 0.05; **p < 0.01; ***p < 0.001.

Supporting H2a, ANOVA results show a statistically significant difference in the self-transcendent emotion of awe scores between groups (see Table 3). Post hoc analyses using the Bonferroni test show that Players reported significantly lower scores of awe than Choreographic Collectives, but not with Other Artists (η_p_^2^ = 0.080). Correlation analyses show that both the number of days and the number of modalities of participation correlated significantly and positively with awe (see Table 4).

Supporting H2b, ANOVA results show there was a statistically significant difference in parochial altruism scores between groups (see Table 3). Post hoc analyses using the Bonferroni test show that Players reported significantly lower scores of parochial altruism than both Choreographic Collectives and Other Artists (η_p_^2^ = 0.121). Correlation analyses show that both the number of days and the number of modalities of participation correlated significantly and positively with parochial altruism (see Table 4).

Partially supporting H2c, ANOVA results show there was a statistically significant difference in community identification scores between groups (see Table 3). Post hoc analyses using the Bonferroni test show that Players have significantly lower scores of Community Identification than Artists (η_p_^2^ = 0.086). Correlation analyses show that the number of days, but not the number of modalities, correlated significantly and positively with community identification (see Table 4).

Failing to find support for H2e, ANOVA results show there were no statistically significant differences in IWAH bond scores between groups (see Table 3). Furthermore, neither the number of modalities, nor the number of days of participation at CNB, were significantly correlated with IWAH bond (see Table 4).

Partially supporting H2f, ANOVA results show there were statistically significant differences in IWAH concern scores between groups (see Table 3). Post hoc analyses using the Bonferroni test show that Choreographic Collectives reported significantly lower scores of IWAH concern than Other Artists (η_p_^2^ = 0.046). In contrast, correlation analysis shows that neither the number of modalities nor the number of days of participation at CNB was significantly correlated with IWAH concern (see Table 4).

### Collective effervescence and its outcomes

Awe, parochial altruism, community identification, national identification, IWAH bond, and IWAH concern intercorrelated positively and significantly (see Table 4). Supporting H3, total collective effervescence correlates with awe, parochial altruism, communal, national and identification with humanity, both bond and concern facets, *r* = 0.55, 0.53, 0.51, 0.37, 0.21 and 0.23, respectively. These outcomes of collective gatherings also correlated positively and significantly with most of the processes of the neo-Durkheimian model. However, effect size or correlations of specific process with all outcomes, were higher for emotional process and self-transcendence experience, medium for social identification process and lowest for behavioral process. Also, correlations were strong for the association between process and proximal identities and lower for the association with IWAH. Specifically, national identification (H3d) and IWAH concern (H3f) were not significantly correlated with shared attention and negative personal emotions. Moreover, IWAH bond (H3e) was not correlated with positive personal emotions, transcendent personal emotions, negative personal emotions, or transcendent experience.

As a complementary way of examining our H3, we conducted a series of six multiple linear regressions using total effervescence, number of days, and number of modalities of participation at CNB as predictors of the outcome variables (see Table 5). Supporting H3a-f, consistently across the six models, total effervescence was a positive and significant predictor of each of the outcome variables, while the number of days and the number of modalities of participation were no longer significant predictors of any of the outcomes.

**Table 5:** Multiple linear regression models with total effervescence, number of days of participation, and number of modalities of participation as predictors of dependent variables.

	Awe	Parochial altruism	Community identification	National identification	IWAH bond	IWAH concern
Total effervescence	0.53 (0.10) *p* < 0.001 [0.58, 0.97]	0.50 (0.08) *p* < 0.001 [0.40, 0.70]	0.50 (0.08) *p* < 0.001 [0.38, 0.66]	0.35 (0.08) *p* < 0.001 [0.22, 0.55]	0.20 (0.11) *p* = 0.013 [0.06, 0.51]	0.21 (0.11) *p* = 0.008 [0.08, 0.51]
# Days of participation	0.06 (0.04) *p* = 0.486 [−0.05, 0.11]	0.06 (0.68) *p* = 0.498 [−0.04, 0.09]	0.15 (0.03) *p* = 0.085 [−0.01, 0.11]	0.16 (0.04) *p* = 0.088 [−0.01, 0.13]	0.11 (0.05) *p* = 0.266 [−0.04, 0.15]	0.10 (0.05) *p* = 0.295 [−0.04, 0.14]
# Modalities of participation	0.05 (0.05) *p* = 0.566 [−0.07, 0.13]	0.12 (0.04) *p* = 0.156 [−0.02, 0.13]	−0.09 (0.04) *p* =. 302 [−0.11, 0.03]	−0.07 (0.04) *p* = 0.469 [−0.11, 0.05]	−0.09 (0.06) *p* = 0.366 [−0.16, 0.06]	−0.02 (0.05) *p* = 0.806 [−0.12, 0.09]
Summary	*R*^2^_Adjusted_: 0.29 *p* < 0.001 *n* = 162	*R*^2^_Adjusted_: 0.29 *p* < 0.001 *n* = 162	*R*^2^_Adjusted_: 0.26 *p* < 0.001 *n* = 162	*R*^2^_Adjusted_: 0.15 *p* < 0.001 *n* = 162	*R*^2^_Adjusted_: 0.03 *p* = 0.040 *n* = 162	*R*^2^_Adjusted_: 0.04 *p* = 0.020 *n* = 162

Total effervescence included all variables in the neo-Durkheimian model except for negative personal emotions.

We further conducted a series of multiple linear regression models on the outcome variables to control for the effect of Age, Socioeconomic Status, Importance Attributed to Religion, and Participants’ Sex (see Appendix B). Consistently across models, total effervescence remained a statistically significant positive predictor of all five of the outcome variables. In these models, there were control variables significantly associated with the outcomes: ideological right was negatively related to parochial altruism, males (compared to females) reported lower scores of community identification, and higher socioeconomic status reported higher scores of IWAH Bond. There were no other statistically significant associations.

### The mediating role of awe

To test H4, we conducted four separate mediation analyses to examine whether Awe mediated the relationship between Total Effervescence and the outcome variables. All analyses used a bootstrapping procedure with 5,000 resamples.

Total Effervescence was significantly associated with Awe in all models (*a* = 0.70, SE = 0.09, *p* < 0.001). In turn, Awe was significantly associated with only two outcomes: parochial altruism (*b* = 0.40, SE = 0.07, *p* < 0.001) and Community Identification (*b* = 0.18, SE = 0.08, *p* = 0.020). Awe was not significantly associated with National Identification (*b* = 0.11, SE = 0.09, *p* = 0.189), IWAH bond (*b* = 0.09, SE = 0.09, *p* = 0.354), and IWAH Concern (*b* = 0.13, SE = 0.09, *p* = 0.167).

Supporting H4a and H4b, the indirect effect of Total Effervescence on the outcome via Awe was significant on two outcomes: parochial altruism (*ab* = 0.28, SE = 0.06, p < 0.001, 95% CI [0.14, 0.48]) and Community Identification (*ab* = 0.13, SE = 0.058, *p* = 0.025, 95% CI [0.17, 0.30]). Contrarily, failing to support H4c-e the indirect effect of Total Effervescence was not statistically significant on National Identification (*ab* = 0.08, SE = 0.06, *p* = 0.195, 95% CI [−0.07, 0.23]); IWAH Bond (*ab* = 0.06, SE = 0.07, *p* = 0.357, 95% CI [−0.07, 0.19]); and IWAH Concern (*ab* = 0.09, SE = 0.07, *p* = 0.172, 95% CI [0.10, 0.32]).

Direct effects (*c′*) of Total Effervescence were significant for parochial altruism (c’ = 0.40, *SE* = 0.10, *p* < 0.001), Community Identification (c’ = 0.53, *SE* = 0.10, *p* < 0.001), and National Identification (c’ = 0.40, *SE* = 0.11, *p* < 0.001). Direct effects of Total effervescence were non-significant on IWAH Bond (c’ = 0.21, *SE* = 0.12, *p* = 0.079) and IWAH Concern (c’ = 0.21, SE = 0.12, *p* = 0.078).

These findings suggest that awe partially explains the positive relationship between Total Effervescence and parochial altruism (H4a) and community identification (H4b). In turn, its effect on National Identification is only direct. Contrary to our expectations, the association between total effervescence and IWAH bond and concern was only significant in its total effects (H4c-e).

## Discussion

The present study aimed to evaluate the association between different forms of participation at CNB, the effects of experiencing the socio-cognitive and emotional processes of collective gatherings, parochial altruism, and social identification. Community and national identification were higher than IWAH. Parochial altruism and all levels of identification correlated positively and significantly. Community, national, and IWAH are intercorrelated positively (Hamer et al., 2021). These findings confirm that higher IWAH and national identities are not opposite. In general, identification with the nation is higher than IWAH, while national identification is notably high among cosmopolitan subjects (Bayram, 2019).

As hypothesized in H1, the less active group or players experienced lower scores of the neo Durkheimian processes model, including positive and transcendent emotions (compared to dancers and other artists), transcendent experience, and total effervescence (compared to dancers exclusively). Similarly, participants who participated in CNB for more days and across more modalities reported higher levels of these processes. The finding that more active groups (dancers and other artists) experience greater proximal and superordinate identification is in line with previous research. Studies show that participants in collective gatherings develop higher levels of social identification as a function of the strength of group connectedness (Xygalatas, 2022), behavioral immersion, and knowledge of heritage value (Ardila Guerrero et al., 2018; Lázaro Ortiz and Jiménez De Madariaga, 2022; Li et al., 2024; Swarbrick and Vuoskoski, 2023).

Results support H1, because more active groups of participants, as well as number of days and modalities, were related to higher total collective effervescence scores, with a medium low effect size, explaining between 5 and 2% of the experience variance. Failing to support at specific process level of collective effervescence H1, neither the groups of participants nor the number of days and modalities were related to some of the model’s processes: situated social identity, shared attention, behavioral synchrony, identity fusion, and emotional synchrony. The exception was that the number of days was positively associated with situated social identification.

As hypothesized in H2, the less active group of players reports lower scores of awe (than dancers), parochial altruism (than both dancers and other artists), and community identification (than artists). Complementarily, awe and parochial altruism were positively correlated with the number of days and modalities of participation, while only the number of days was positively correlated with community and national identification. We attribute these results to the nature of emotional engagement and expression inherent in different art forms. Particularly, the perception of being immersed in a context of social change leads to the development of an emerging identity that creates multiple sources of identification with others (Chayinska et al., 2017; Hopkins et al., 2016; Mason, 2023; Sabucedo et al., 2018; Van Breen et al., 2017; van Zomeren et al., 2018; Zamudio et al., 2022; Zamudio and Montero-Lopéz, 2021).

Failing to support H2, results show no differences between groups of participants in national identification, IWAH bond, or IWAH concern. Similarly, the number of days and modalities of participation were not related to the IWAH bond or IWAH concern. These results suggest that it is not the frequency and form of participation at a collective gathering that is associated with its outcome on superordinate identification. Instead, it is the perceived quality as experienced by those who take part in it. In line with the neo-Durkheimian model of collective gatherings, the cognitive and emotional processes leading to collective effervescence predict the outcomes of collective gatherings, while the mere involvement is not enough to produce such effects, despite how frequent or prolonged it is (Rimé and Páez, 2023). Moreover, following the Common Ingroup Identity Model (Gaetner and Dovidio, 2012), the prominence of a more inclusive higher identity category, containing both in-group and outgroup identities, may reduce intergroup stereotypes and foster harmony between conflicting groups. However, at CNB, the multiple identities enacted within the event fail to produce a considerable effect on the most superordinate identity with all of humanity.

As hypothesized in H3, awe, parochial altruism, community identification, national identification, and IWAH concern and bond were consistently associated with total collective effervescence, and with most of the neo-Durkheimian model processes. Moreover, the significant association between total effervescence and all the outcome variables remained significant even when the number of days of participation and the number of modalities of participation were entered into multiple regression models. Key to our findings, and consistent with previous studies, awe had the largest effect size in the association between collective gathering processes on identification with all humanity (Pizarro et al., 2021a, 2021b; Zabala et al., 2023). The experience of self-transcendent emotions increases the degree to which a group member focuses attention on collective issues, symbols, and values, thereby increasing identification with broader social categories (Pizarro et al., 2018).

Additionally, consistently across variables, personal negative emotions correlated with low community identification. Negative emotions are less relevant or play an inhibitory role in festive collective gatherings, likely due to their positive valence, despite the significant role of anger and sadness in the outcomes of demonstrations and mourning rituals, respectively (Pizarro et al., 2022).

Despite the finding that total collective effervescence is correlated with both IWAH bond and IWAH concern, some of the processes of the neo-Durkheimian model were not correlated with the IWAH factors. IWAH bond was correlated with identity fusion, emotional synchrony, and effervescence, suggesting that process of situated social identity, behavioral synchrony, and shared attention are less relevant for the superordinate bond (Rimé and Páez, 2023). Additionally, among the processes of the neo-Durkheimian model, IWAH concern was not correlated with identity fusion exclusively. However, even if some processes did not correlate with outcomes, the global score of collective effervescence correlates strongly with IWAH facets. We consider that higher statistical power would be able to identify a significant correlation between IWAH concern and identity fusion, although with a moderate effect size. In the same line, effect sizes of the association between the neo-Durkheimian model processes and community and national identification are larger than the IWAH factors, reinforcing the proposition that the more superordinate the reference group, the smaller the association between these processes and social identification (Huddy, 2023). Results support that quality, more than intensity of participation in collective rituals such as CNB, enhances local group identities and parochial altruism. Also, to a lesser extent, quality enhances superordinate identity with humankind. Results also suggest that carnivals are less effective in promoting IWAH, than in celebrating a local identity (Hamer et al., 2021; Wlodarczyk et al., 2022; Zabala et al., 2023).

Results failed to support H4. On the one hand, awe correlates with proximal identities (community and national), but also with identification with all of humanity. In this sense, awe is associated with beliefs of transcendence (Keltner, 2023), such as identification with humanity. However, the association was stronger with proximal identities than with all of humanity. On the other hand, awe partially mediated the effect of total effervescence on parochial altruism and community identification. However, awe did not mediate the effect of total effervescence on more superordinate levels of identification: national identification, IWAH bond, nor IWAH concern. One likely reason for these results could be that greater participation in heritage events such as CNB reinforces awe related to identification with the groups more represented in the event, such as the community, rather than with broader categories such as identification with humanity (Chng and Narayanan, 2017; Grant, 2014).

### Limitations and future directions

The present study had important limitations that must be considered when evaluating its findings. First, the sample size was small considering the population of artists and musicians performing during the CNB, but time and resource constraints during data collection made it difficult to obtain more responses. Future research should develop a data collection strategy that allows for a larger sample size or a different sampling strategy to further support the hypotheses tested in this study.

Second, data collection took place in August, which is a period of intense collective activities before auditions for the event, especially by choreographic collectives, and to a lesser extent by other artists. Therefore, the effect on social identity and prosocial behavior of participation in the previous CNB (between December and January) might be decreasing, while the effect of preparation for the next CNB might be increasing. Another limitation of the study is its retrospective nature: the survey on the CNB was conducted 7 month after the event. This probably increases memory bias. Longitudinal approaches applying pretest, posttest and follow up measures, will provide key information to understand the consequences of participation in heritage events for an overall identity, as well as the duration of that effect.

Third, there is a need for qualitative and mixed approaches to the social identities that develop in the CNB, as in a heritage that deals explicitly with ethnic identification; these results will shed light on the intergroup experiences of participants. Not only performers, but also carnival players and workers experience intergroup contact during the event.

Fourth, it is also important to explore the relative importance of the different processes of collective effervescence in the future and confirm whether the results of this study (higher effect size emotional process and self-transcendence experience, medium for social identification process and lowest for behavioral process) can be generalized.

## Data Availability

The raw data supporting the conclusions of this article will be made available by the authors, without undue reservation.
