# Supplementary material for: Ritual’s collective effervescence, awe, and social identity: psychosocial effects of the Pasto carnival
Source: Front Psychol. 2025 Nov 11;16:1566499. doi: 10.3389/fpsyg.2025.1566499 (PMC12643837; doi:10.3389/fpsyg.2025.1566499)
Supplement: Supplementary file 1 [file Table_1.DOCX]

**Appendix A. Measures**

**Age:** _______.

**Gender Identity:**

1. Male
2. Female
3. Other

**Country of Birth:** _________________________________________.
**State/Province of Birth:** ____________________________________.

Did you participate in the 2023 Carnival?

1. Yes
2. No

If yes, how? (Select all that apply):

- 1. Individual/couple/group costume
  2. Comparsa (folkloric dance group)
  3. Murga (musical parade group)
  4. Choreographic collective
  5. Non-motorized float
  6. Motorized float
  7. Played with talcum/cosmetics on streets
  8. Participated in Arcoíris en el Asfalto
  9. Attended the antique car parade
  10. Attended the años viejos parade
  11. Attended concerts at Plaza del Carnaval or Plaza de Nariño
  12. Carnival organizer (e.g., Corpocarnaval)
  13. Member of a participating group
  14. Press (local/national/international)
  15. Attendee only

Participation Days (2023): (Select all that apply)

1. December 28.
2. December 29
3. December 30
4. December 31
5. January 2
6. January 3
7. January 4
8. January 5
9. January 6
10. January 7

To what extent do you disagree or agree with the following statements? Please use the following scale: Not at all = 1 2 3 3 4 4 5 6 7 = Very much.

| I feel a bond with the people who participate in the Carnaval de Negros y Blancos. | 1 | 2 | 3 | 4 | 5 | 6 | 7 |
| --- | --- | --- | --- | --- | --- | --- | --- |
| I feel solidarity with the people who participate in the Carnaval de Negros y Blancos. | 1 | 2 | 3 | 4 | 5 | 6 | 7 |
| I feel a moral commitment to the people who participate in the Carnaval de Negros y Blancos. | 1 | 2 | 3 | 4 | 5 | 6 | 7 |

**The “Carnaval de Negros y Blancos” is festive act about heritage and “mestizaje” that is nourished by multiple manifestations and expressions, creating a festive period for diverse human groups, and social sectors to participate massively”.**

How important is it for you...? Please answer using the following scale: Not at all important = 1 2 3 3 4 5 6 7 = Very important.

| Transmit the knowledge of the carnival from generation to generation. | 1 | 2 | 3 | 4 | 5 | 6 | 7 |
| --- | --- | --- | --- | --- | --- | --- | --- |
| Promote the memory of myths, legends, characters, historical facts, and emblematic elements of my community | 1 | 2 | 3 | 4 | 5 | 6 | 7 |
| To diminish the differences between cultural and ethnic groups and sectors | 1 | 2 | 3 | 4 | 5 | 6 | 7 |
| Increase the participation of members of my community in public decision making. | 1 | 2 | 3 | 4 | 5 | 6 | 7 |
| Promote respect for the artisan, the city, the culture, and the land. | 1 | 2 | 3 | 4 | 5 | 6 | 7 |
| To link tourists and visitors to the traditions of my community. | 1 | 2 | 3 | 4 | 5 | 6 | 7 |

**At this point, you will have realized that you may have taken part in collective activities of the Carnaval de Negros y Blancos 2023 as a participant (float, collective, murga, comparsa, costume, antique car parade, parade of años viejos), as an attendee of the events (concert, game with talcum powder and cosmetic), or as an organizer of the carnival (Corpocarnaval, Mayor's Office, Governor's Office). The following questions take into account the three forms of participation.**

Please indicate how often you had the following experiences during the Carnaval de Negros y Blancos 2023. Please answer using the following scale: Strongly Disagree = 1 2 3 3 4 4 5 6 7 = Strongly Agree.

| I felt identified with the other members of the crowd who were in the collective activity. | 1 | 2 | 3 | 4 | 5 | 6 | 7 |
| --- | --- | --- | --- | --- | --- | --- | --- |
| I felt like the other people who were in the collective activity. | 1 | 2 | 3 | 4 | 5 | 6 | 7 |
| I felt strong bonds with other people who were in the collective activity. | 1 | 2 | 3 | 4 | 5 | 6 | 7 |

Indicate how often you had the following experiences during the Carnaval de Negros y Blancos 2023. Please answer using the following scale: None = 1 2 3 3 4 5 6 7 = A lot.

| People who participated in the event attended to the same stimuli, symbols, objects, or events. | 1 | 2 | 3 | 4 | 5 | 6 | 7 |
| --- | --- | --- | --- | --- | --- | --- | --- |
| People simultaneously concentrated or focused their attention on the same stimuli. | 1 | 2 | 3 | 4 | 5 | 6 | 7 |
| Las personas atendían al mismo tiempo a ciertos aspectos del evento. | 1 | 2 | 3 | 4 | 5 | 6 | 7 |
| People attended to certain aspects of the event at the same time. | 1 | 2 | 3 | 4 | 5 | 6 | 7 |
| The people who participated in the event carried out a coordinated activity. They all did the same thing at the same time, such as clapping, dancing, praying, cheering. | 1 | 2 | 3 | 4 | 5 | 6 | 7 |
| They performed a shared or convergent activity in time. | 1 | 2 | 3 | 4 | 5 | 6 | 7 |
| They acted in harmony. | 1 | 2 | 3 | 4 | 5 | 6 | 7 |

Indicate how often you had the following experiences during the Carnaval de Negros y Blancos 2023. Answer thinking about the **people who were in the collective activity** and please use the following scale: None = 1 2 3 3 4 5 6 7 = A lot.

| We all acted as one person. | 1 | 2 | 3 | 4 | 5 | 6 | 7 |
| --- | --- | --- | --- | --- | --- | --- | --- |
| It felt like we were one person. | 1 | 2 | 3 | 4 | 5 | 6 | 7 |
| We felt that we were a whole. | 1 | 2 | 3 | 4 | 5 | 6 | 7 |
| We felt more sensitive to the emotions and feelings of other people experiencing the same thing. | 1 | 2 | 3 | 4 | 5 | 6 | 7 |
| We felt a strong shared emotion. | 1 | 2 | 3 | 4 | 5 | 6 | 7 |
| We felt stronger emotions than what we usually experience. | 1 | 2 | 3 | 4 | 5 | 6 | 7 |

During the Carnaval de Negros y Blancos 2023, to what extent did you have the following thoughts.

**Think about the people who were in the collective activity** you took part in **as your group**, and please use the following scale: Strongly Disagree = 1 2 3 3 4 4 5 6 7 = Strongly Agree.

| My group was me. | 1 | 2 | 3 | 4 | 5 | 6 | 7 |
| --- | --- | --- | --- | --- | --- | --- | --- |
| My group and I were one. | 1 | 2 | 3 | 4 | 5 | 6 | 7 |
| I felt immersed in my group. | 1 | 2 | 3 | 4 | 5 | 6 | 7 |
| I felt a strong emotional bond with my group. | 1 | 2 | 3 | 4 | 5 | 6 | 7 |
| I made my group stronger. | 1 | 2 | 3 | 4 | 5 | 6 | 7 |
| I wanted to do more for my group than any other group member would want to do. | 1 | 2 | 3 | 4 | 5 | 6 | 7 |

During the Carnaval de Negros y Blancos 2023 in which I participated, or witnessed it directly in person, or through the mass media, I felt.... Please answer using the following scale: Not at all = 1 2 3 3 4 5 6 7 = Very much.

| Accomplished. | 1 | 2 | 3 | 4 | 5 | 6 | 7 |
| --- | --- | --- | --- | --- | --- | --- | --- |
| Happy. | 1 | 2 | 3 | 4 | 5 | 6 | 7 |
| Alive, active. | 1 | 2 | 3 | 4 | 5 | 6 | 7 |

In the Carnaval de Negros y Blancos 2023 to what extent did you feel or did you feel...? Please answer using the following scale: Not at all = 1 2 3 3 4 5 6 7 = Very much.

| Excited, moved. | 1 | 2 | 3 | 4 | 5 | 6 | 7 |
| --- | --- | --- | --- | --- | --- | --- | --- |
| Amazed, astonished, amazed, awed by something great. | 1 | 2 | 3 | 4 | 5 | 6 | 7 |
| Morally inspired, enlightened, enthusiastic. | 1 | 2 | 3 | 4 | 5 | 6 | 7 |
| Love, closeness, trust. | 1 | 2 | 3 | 4 | 5 | 6 | 7 |
| Solidarity, supportive of others. | 1 | 2 | 3 | 4 | 5 | 6 | 7 |
| Hopeful, optimistic, encouraged. | 1 | 2 | 3 | 4 | 5 | 6 | 7 |
| Angry, irritated, annoyed. | 1 | 2 | 3 | 4 | 5 | 6 | 7 |
| Stressed, nervous or overwhelmed. | 1 | 2 | 3 | 4 | 5 | 6 | 7 |
| Sad, discouraged or unhappy. | 1 | 2 | 3 | 4 | 5 | 6 | 7 |

Indicate how often you had the following experiences during the Carnaval de Negros y Blancos 2023. Please answer using the following scale: Strongly Disagree = 1 2 3 3 4 4 5 6 7 = Strongly Agree.

| I felt that the event had an important purpose or objective. | 1 | 2 | 3 | 4 | 5 | 6 | 7 |
| --- | --- | --- | --- | --- | --- | --- | --- |
| I felt that there was something associated with values and ideals in the event. | 1 | 2 | 3 | 4 | 5 | 6 | 7 |
| I felt that there was something special about the event. | 1 | 2 | 3 | 4 | 5 | 6 | 7 |
| I felt as if the event had changed me in some way. | 1 | 2 | 3 | 4 | 5 | 6 | 7 |

Indicate how often you had the following thoughts or sensations during the Carnaval de Negros y Blancos 2023. Answer thinking about the people who were in the collective activity in which you participated. Please use the following scale: Never = 1 2 3 3 4 5 6 7 = Always.

| We were sure of what we wanted to do. | 1 | 2 | 3 | 4 | 5 | 6 | 7 |
| --- | --- | --- | --- | --- | --- | --- | --- |
| We were totally focused on what we were doing. | 1 | 2 | 3 | 4 | 5 | 6 | 7 |
| We felt we could control what we were doing. | 1 | 2 | 3 | 4 | 5 | 6 | 7 |
| We all found the experience we had together very valuable and comforting. | 1 | 2 | 3 | 4 | 5 | 6 | 7 |
| We felt that we were good enough to meet the challenge or difficulty of the situation. | 1 | 2 | 3 | 4 | 5 | 6 | 7 |
| We were confident that, at the time, we were doing well. | 1 | 2 | 3 | 4 | 5 | 6 | 7 |

Indicate how often you had the following experiences during the Carnaval de Negros y Blancos 2023 in which you participated. Please answer using the following scale: Strongly Disagree = 1 2 3 3 4 5 6 = Strongly Agree.

| I had a combination of emotions that probably other people have never experienced. | 1 | 2 | 3 | 4 | 5 | 6 |
| --- | --- | --- | --- | --- | --- | --- |
| I was able to experience a variety of different emotions at the same time. | 1 | 2 | 3 | 4 | 5 | 6 |
| I had emotional experiences that could be considered unusual or out of the ordinary. | 1 | 2 | 3 | 4 | 5 | 6 |
| I was able to experience a large number of different emotions. | 1 | 2 | 3 | 4 | 5 | 6 |

To what degree would you be willing to engage in the following activities in favor of the collective, movement or group with which you participated in the Carnaval de Negros y Blancos 2023? Please answer using the following scale: Not at all = 1 2 3 4 5 = Very much.

| Participate in future versions of the Carnaval de Negros y Blancos. | 1 | 2 | 3 | 4 | 5 |
| --- | --- | --- | --- | --- | --- |
| Provide your cell phone number, email, or WhatsApp to receive information about future events of the Carnaval de Negros y Blancos. | 1 | 2 | 3 | 4 | 5 |
| Commit 2 hours per week to collaborate with a group that participates in the Carnaval de Negros y Blancos. | 1 | 2 | 3 | 4 | 5 |
| Wear an emblem or symbol (badge, clothing, etc.) of the Carnaval de Negros y Blancos. | 1 | 2 | 3 | 4 | 5 |
| Undergo training to become an agent for the defense of the region's cultural heritage. | 1 | 2 | 3 | 4 | 5 |

How close do you feel to each of the following groups? Please check the option that best represents your feelings.... Not at all close = 1 2 3 4 5 = Very close.

| My community. | 1 | 2 | 3 | 4 | 5 |
| --- | --- | --- | --- | --- | --- |
| My nation/country. | 1 | 2 | 3 | 4 | 5 |
| People from all over the world. | 1 | 2 | 3 | 4 | 5 |

How much would you say you have **in common** with the following groups? Almost nothing in common = 1 2 3 4 5 = A lot in common.

| My community | 1 | 2 | 3 | 4 | 5 |
| --- | --- | --- | --- | --- | --- |
| My nation/country. | 1 | 2 | 3 | 4 | 5 |
| People from all over the world. | 1 | 2 | 3 | 4 | 5 |

How much would you say you care (feel bothered or want to help) when bad things happen to...? Not at all = 1 2 3 4 5= Very much.

| My community | 1 | 2 | 3 | 4 | 5 |
| --- | --- | --- | --- | --- | --- |
| My nation/country. | 1 | 2 | 3 | 4 | 5 |
| People from all over the world. | 1 | 2 | 3 | 4 | 5 |

If the need comes up, how much would you like to help the following groups. Not at all = 1 2 3 4 5 = Very much.

| My community | 1 | 2 | 3 | 4 | 5 |
| --- | --- | --- | --- | --- | --- |
| My nation/country. | 1 | 2 | 3 | 4 | 5 |
| People from all over the world. | 1 | 2 | 3 | 4 | 5 |

**Feeling veneration, wonder and/or awe, is the emotion felt before something that produces a sensation of “immensity” and has great “richness” (such as an impressive natural landscape, a monument, or impressive construction, or a grandiose artistic, cultural, religious, ideological, labor action). People who report this emotion often indicate that they feel they are “small” and part of something bigger than themselves.**

**Please respond using the following scale: Not at all = 1 2 3 3 4 5 6 7 = Very much.**

| How often do you generally feel awe-struck respect, veneration and/or awe-struck wonder? | 1 | 2 | 3 | 4 | 5 | 6 | 7 |
| --- | --- | --- | --- | --- | --- | --- | --- |

Using the same scale: Not at all = 1 2 3 4 5 6 7 = Very much, indicate how intensely you experienced it in the Carnaval de Negros y Blancos 2023.

| I felt I was in the presence of something great. | 1 | 2 | 3 | 4 | 5 | 6 | 7 |
| --- | --- | --- | --- | --- | --- | --- | --- |
| I was deeply moved by the experience. | 1 | 2 | 3 | 4 | 5 | 6 | 7 |
| I felt small in the presence of something great. | 1 | 2 | 3 | 4 | 5 | 6 | 7 |
| I felt that time slowed down, things slowed down for a moment. | 1 | 2 | 3 | 4 | 5 | 6 | 7 |
| I wanted to be part of something bigger than myself. | 1 | 2 | 3 | 4 | 5 | 6 | 7 |
| I felt that life and the world made sense. | 1 | 2 | 3 | 4 | 5 | 6 | 7 |

In which socio-economic stratum do you live?

| 1 | 2 | 3 | 4 | 5 | 6 |
| --- | --- | --- | --- | --- | --- |

If we talk about gender, how do you identify yourself?

1. Masculine.

2. Feminine.

3. Other.

In matters of politics, people talk about right and left, where would you place yourself on this continuum: Left = 1 2 3 3 4 5 6 7 = Right?

| 1 | 2 | 3 | 4 | 5 | 6 | 7 |
| --- | --- | --- | --- | --- | --- | --- |

How important is religion in your life? Place yourself on a continuum where Not at all important = 1 2 3 4 = Very important.

| 1 | 2 | 3 | 4 |
| --- | --- | --- | --- |

Do you consider yourself a white, mestizo, indigenous, black, mulatto, or other person?

1. White.

2. Mestizo.

3. Indigenous.

4. Black.

5. Mulatto.

6. Other.

Please indicate your marital status.

1. Single.

2. Married.

3. I live with a partner.

4. Separated.

5. Divorced.

6. Widowed.

7. Other.

What is your main occupation? Are you currently:

1. working.

2. Not working at the moment, but have a job.

3. You are actively looking for work.

4. student.

5. Engaged in domestic chores.

6. Permanently unable to work.

7. retired or pensioned.

8. not working and are not looking for work.

What is your highest level of education?

1. None.

2. Primary school.

3. High school.

4. College.

5. Higher non-university.

6. Postgraduate.

7. Other.
