# Supplementary material for: Ritual’s collective effervescence, awe, and social identity: psychosocial effects of the Pasto carnival
Source: Front Psychol. 2025 Nov 11;16:1566499. doi: 10.3389/fpsyg.2025.1566499 (PMC12643837; doi:10.3389/fpsyg.2025.1566499)
Supplement: Supplementary file 2 [file Table_2.DOCX]

**Appendix A**

Multiple Linear Regression Models of Total Effervescence with Control Variables on Outcome Variables

**B.1. Parochial Altruism**

***Controlled by Age***

|  | | | | | | **95% CI** | |
| --- | --- | --- | --- | --- | --- | --- | --- |
|  | **B** | **SE** | **β** | **t** | **p** | **Lower** | **Upper** |
| (Intercept) | 0.785 | 0.446 |  | 1.759 | 0.081 | -0.096 | 1.667 |
| Total Effervescence | 0.569 | 0.075 | 0.513 | 7.575 | < .001 | 0.421 | 0.717 |
| Age | 0.007 | 0.005 | 0.095 | 1.411 | 0.160 | -0.003 | 0.017 |

***Controlled by Socioeconomic Status***

|  | | | | | | **95% CI** | |
| --- | --- | --- | --- | --- | --- | --- | --- |
|  | **B** | **SE** | **β** | **t** | **p** | **Lower** | **Upper** |
| (Intercept) | 1.050 | 0.448 |  | 2.346 | 0.020 | 0.166 | 1.934 |
| Total Effervescence | 0.589 | 0.074 | 0.531 | 7.926 | < .001 | 0.442 | 0.736 |
| Socioeconomic Status | -0.072 | 0.044 | -0.109 | -1.626 | 0.106 | -0.160 | 0.016 |

***Controlled by Ideological Orientation***

|  | | | | | | **95% CI** | |
| --- | --- | --- | --- | --- | --- | --- | --- |
|  | **B** | **SE** | **β** | **t** | **p** | **Lower** | **Upper** |
| (Intercept) | 1.047 | 0.437 |  | 2.396 | 0.018 | 0.184 | 1.910 |
| Total Effervescence | 0.609 | 0.074 | 0.549 | 8.217 | < .001 | 0.463 | 0.756 |
| Ideological Orientation | -0.081 | 0.032 | -0.168 | -2.518 | 0.013 | -0.144 | -0.017 |

***Controlled by Importance of Religion***

|  | | | | | | **95% CI** | |
| --- | --- | --- | --- | --- | --- | --- | --- |
|  | **B** | **SE** | **β** | **t** | **p** | **Lower** | **Upper** |
| (Intercept) | 1.000 | 0.450 |  | 2.220 | 0.028 | 0.110 | 1.890 |
| Total Effervescence | 0.595 | 0.075 | 0.536 | 7.885 | < .001 | 0.446 | 0.744 |
| Importance of Religion | -0.057 | 0.055 | -0.071 | -1.045 | 0.298 | -0.166 | 0.051 |

***Controlled by Sex***

|  | | | | | | **95% CI** | |
| --- | --- | --- | --- | --- | --- | --- | --- |
|  | **B** | **SE** | **β** | **t** | **p** | **Lower** | **Upper** |
| (Intercept) | 0.934 | 0.457 |  | 2.043 | 0.043 | 0.031 | 1.837 |
| Total Effervescence | 0.580 | 0.075 | 0.523 | 7.687 | < .001 | 0.431 | 0.729 |
| Sex (Male) | -0.034 | 0.117 | -0.020 | -0.288 | 0.774 | -0.265 | 0.197 |

**B.2. Community Identification**

***Controlled by Age***

|  | | | | | | **95% CI** | |
| --- | --- | --- | --- | --- | --- | --- | --- |
|  | **B** | **SE** | **β** | **t** | **p** | **Lower** | **Upper** |
| (Intercept) | 1.067 | 0.422 |  | 2.528 | 0.012 | 0.233 | 1.900 |
| Total Effervescence | 0.525 | 0.071 | 0.507 | 7.396 | < .001 | 0.385 | 0.665 |
| Age | 0.003 | 0.005 | 0.051 | 0.741 | 0.460 | -0.006 | 0.013 |

***Controlled by Socioeconomic Status***

|  | | | | | | **95% CI** | |
| --- | --- | --- | --- | --- | --- | --- | --- |
|  | **B** | **SE** | **β** | **t** | **p** | **Lower** | **Upper** |
| (Intercept) | 1.118 | 0.425 |  | 2.633 | 0.009 | 0.280 | 1.957 |
| Total Effervescence | 0.532 | 0.071 | 0.514 | 7.541 | < .001 | 0.393 | 0.671 |
| Socioeconomic Status | 0.002 | 0.042 | 0.004 | 0.059 | 0.953 | -0.081 | 0.086 |

***Controlled by Ideological Orientation***

|  | | | | | | **95% CI** | |
| --- | --- | --- | --- | --- | --- | --- | --- |
|  | **B** | **SE** | **β** | **t** | **p** | **Lower** | **Upper** |
| (Intercept) | 1.164 | 0.419 |  | 2.780 | 0.006 | 0.337 | 1.991 |
| Total Effervescence | 0.539 | 0.071 | 0.521 | 7.593 | < .001 | 0.399 | 0.680 |
| Ideological Orientation | -0.022 | 0.031 | -0.050 | -0.722 | 0.471 | -0.083 | 0.039 |

***Controlled by Importance of Religion***

|  | | | | | | **95% CI** | |
| --- | --- | --- | --- | --- | --- | --- | --- |
|  | **B** | **SE** | **β** | **t** | **p** | **Lower** | **Upper** |
| (Intercept) | 1.167 | 0.425 |  | 2.746 | 0.007 | 0.328 | 2.007 |
| Total Effervescence | 0.537 | 0.071 | 0.519 | 7.549 | < .001 | 0.397 | 0.678 |
| Importance of Religion | -0.025 | 0.052 | -0.033 | -0.483 | 0.629 | -0.128 | 0.077 |

***Controlled by Sex***

|  | | | | | | **95% CI** | |
| --- | --- | --- | --- | --- | --- | --- | --- |
|  | **B** | **SE** | **β** | **t** | **p** | **Lower** | **Upper** |
| (Intercept) | 1.397 | 0.422 |  | 3.308 | 0.001 | 0.563 | 2.231 |
| Total Effervescence | 0.509 | 0.070 | 0.492 | 7.306 | < .001 | 0.372 | 0.647 |
| Sex (Male) | -0.269 | 0.108 | -0.168 | -2.494 | 0.014 | -0.483 | -0.056 |

**B.3. National Identification**

***Controlled by Age***

|  | | | | | | **95% CI** | |
| --- | --- | --- | --- | --- | --- | --- | --- |
|  | **B** | **SE** | **β** | **t** | **p** | **Lower** | **Upper** |
| (Intercept) | 1.491 | 0.489 |  | 3.047 | 0.003 | 0.525 | 2.457 |
| Total Effervescence | 0.398 | 0.082 | 0.358 | 4.834 | < .001 | 0.235 | 0.560 |
| Age | 0.006 | 0.005 | 0.081 | 1.088 | 0.278 | -0.005 | 0.016 |

***Controlled by Socioeconomic Status***

|  | | | | | | **95% CI** | |
| --- | --- | --- | --- | --- | --- | --- | --- |
|  | **B** | **SE** | **β** | **t** | **p** | **Lower** | **Upper** |
| (Intercept) | 1.491 | 0.492 |  | 3.030 | 0.003 | 0.519 | 2.463 |
| Total Effervescence | 0.406 | 0.082 | 0.366 | 4.970 | < .001 | 0.245 | 0.567 |
| Socioeconomic Status | 0.047 | 0.049 | 0.070 | 0.957 | 0.340 | -0.050 | 0.143 |

***Controlled by Ideological Orientation***

|  | | | | | | **95% CI** | |
| --- | --- | --- | --- | --- | --- | --- | --- |
|  | **B** | **SE** | **β** | **t** | **p** | **Lower** | **Upper** |
| (Intercept) | 1.561 | 0.487 |  | 3.205 | 0.002 | 0.599 | 2.523 |
| Total Effervescence | 0.405 | 0.083 | 0.365 | 4.906 | < .001 | 0.242 | 0.568 |
| Ideological Orientation | 0.015 | 0.036 | 0.031 | 0.415 | 0.678 | -0.056 | 0.086 |

***Controlled by Importance of Religion***

|  | | | | | | **95% CI** | |
| --- | --- | --- | --- | --- | --- | --- | --- |
|  | **B** | **SE** | **β** | **t** | **p** | **Lower** | **Upper** |
| (Intercept) | 1.531 | 0.494 |  | 3.100 | 0.002 | 0.556 | 2.506 |
| Total Effervescence | 0.403 | 0.083 | 0.363 | 4.883 | < .001 | 0.240 | 0.567 |
| Importance of Religion | 0.033 | 0.060 | 0.041 | 0.545 | 0.586 | -0.086 | 0.152 |

***Controlled by Sex***

|  | | | | | | **95% CI** | |
| --- | --- | --- | --- | --- | --- | --- | --- |
|  | **B** | **SE** | **β** | **t** | **p** | **Lower** | **Upper** |
| (Intercept) | 1.727 | 0.498 |  | 3.467 | < .001 | 0.743 | 2.711 |
| Total Effervescence | 0.398 | 0.082 | 0.359 | 4.843 | < .001 | 0.236 | 0.561 |
| Sex (Male) | -0.137 | 0.127 | -0.080 | -1.078 | 0.283 | -0.389 | 0.114 |

**B.4. IWAH Bond**

***Controlled by Age***

|  | | | | | | **95% CI** | |
| --- | --- | --- | --- | --- | --- | --- | --- |
|  | **B** | **SE** | **β** | **t** | **p** | **Lower** | **Upper** |
| (Intercept) | 1.350 | 0.662 |  | 2.041 | 0.043 | 0.044 | 2.657 |
| Total Effervescence | 0.291 | 0.111 | 0.205 | 2.613 | 0.010 | 0.071 | 0.510 |
| Age | 0.002 | 0.007 | 0.020 | 0.260 | 0.795 | -0.012 | 0.016 |

***Controlled by Socioeconomic Status***

|  | | | | | | **95% CI** | |
| --- | --- | --- | --- | --- | --- | --- | --- |
|  | **B** | **SE** | **β** | **t** | **p** | **Lower** | **Upper** |
| (Intercept) | 1.060 | 0.653 |  | 1.623 | 0.106 | -0.230 | 2.351 |
| Total Effervescence | 0.282 | 0.108 | 0.198 | 2.596 | 0.010 | 0.067 | 0.496 |
| Socioeconomic Status | 0.155 | 0.065 | 0.182 | 2.385 | 0.018 | 0.027 | 0.283 |

***Controlled by Ideological Orientation***

|  | | | | | | **95% CI** | |
| --- | --- | --- | --- | --- | --- | --- | --- |
|  | **B** | **SE** | **β** | **t** | **p** | **Lower** | **Upper** |
| (Intercept) | 1.297 | 0.655 |  | 1.982 | 0.049 | 0.004 | 2.590 |
| Total Effervescence | 0.280 | 0.111 | 0.197 | 2.520 | 0.013 | 0.060 | 0.499 |
| Ideological Orientation | 0.047 | 0.048 | 0.075 | 0.966 | 0.335 | -0.049 | 0.142 |

***Controlled by Importance of Religion***

|  | | | | | | **95% CI** | |
| --- | --- | --- | --- | --- | --- | --- | --- |
|  | **B** | **SE** | **β** | **t** | **p** | **Lower** | **Upper** |
| (Intercept) | 1.240 | 0.664 |  | 1.869 | 0.063 | -0.070 | 2.551 |
| Total Effervescence | 0.279 | 0.111 | 0.196 | 2.507 | 0.013 | 0.059 | 0.498 |
| Importance of Religion | 0.081 | 0.081 | 0.078 | 1.001 | 0.318 | -0.079 | 0.241 |

***Controlled by Sex***

|  | | | | | | **95% CI** | |
| --- | --- | --- | --- | --- | --- | --- | --- |
|  | **B** | **SE** | **β** | **t** | **p** | **Lower** | **Upper** |
| (Intercept) | 1.277 | 0.673 |  | 1.898 | 0.060 | -0.052 | 2.606 |
| Total Effervescence | 0.303 | 0.111 | 0.213 | 2.731 | 0.007 | 0.084 | 0.523 |
| Sex (Male) | 0.103 | 0.172 | 0.047 | 0.600 | 0.549 | -0.237 | 0.443 |

**B.5. IWAH Concern**

***Controlled by Age***

|  | | | | | | **95% CI** | |
| --- | --- | --- | --- | --- | --- | --- | --- |
|  | **B** | **SE** | **β** | **t** | **p** | **Lower** | **Upper** |
| (Intercept) | 1.821 | 0.640 |  | 2.846 | 0.005 | 0.557 | 3.084 |
| Total Effervescence | 0.309 | 0.108 | 0.224 | 2.871 | 0.005 | 0.096 | 0.522 |
| Age | 0.003 | 0.007 | 0.035 | 0.452 | 0.652 | -0.011 | 0.017 |

***Controlled by Socioeconomic Status***

|  | | | | | | **95% CI** | |
| --- | --- | --- | --- | --- | --- | --- | --- |
|  | **B** | **SE** | **β** | **t** | **p** | **Lower** | **Upper** |
| (Intercept) | 1.745 | 0.641 |  | 2.721 | 0.007 | 0.478 | 3.012 |
| Total Effervescence | 0.310 | 0.107 | 0.225 | 2.914 | 0.004 | 0.100 | 0.521 |
| Socioeconomic Status | 0.062 | 0.064 | 0.075 | 0.971 | 0.333 | -0.064 | 0.188 |

***Controlled by Ideological Orientation***

|  | | | | | | **95% CI** | |
| --- | --- | --- | --- | --- | --- | --- | --- |
|  | **B** | **SE** | **β** | **t** | **p** | **Lower** | **Upper** |
| (Intercept) | 1.848 | 0.635 |  | 2.910 | 0.004 | 0.594 | 3.102 |
| Total Effervescence | 0.311 | 0.108 | 0.225 | 2.888 | 0.004 | 0.098 | 0.524 |
| Ideological Orientation | 0.014 | 0.047 | 0.023 | 0.301 | 0.764 | -0.078 | 0.106 |

***Controlled by Importance of Religion***

|  | | | | | | **95% CI** | |
| --- | --- | --- | --- | --- | --- | --- | --- |
|  | **B** | **SE** | **β** | **t** | **p** | **Lower** | **Upper** |
| (Intercept) | 1.972 | 0.643 |  | 3.065 | 0.003 | 0.701 | 3.242 |
| Total Effervescence | 0.327 | 0.108 | 0.236 | 3.036 | 0.003 | 0.114 | 0.539 |
| Importance of Religion | -0.056 | 0.079 | -0.056 | -0.718 | 0.474 | -0.212 | 0.099 |

***Controlled by Sex***

|  | | | | | | **95% CI** | |
| --- | --- | --- | --- | --- | --- | --- | --- |
|  | **B** | **SE** | **β** | **t** | **p** | **Lower** | **Upper** |
| (Intercept) | 2.060 | 0.649 |  | 3.173 | 0.002 | 0.778 | 3.342 |
| Total Effervescence | 0.300 | 0.107 | 0.217 | 2.798 | 0.006 | 0.088 | 0.512 |
| Sex (Male) | -0.184 | 0.166 | -0.086 | -1.107 | 0.270 | -0.512 | 0.144 |

**B.6. Mulitple Linear Regressions With All Controls as Predictors**

***Coefficients for Parochial Altruism (Adj. R^2^ = .023)***

|  | | | | | | **95% CI** | |
| --- | --- | --- | --- | --- | --- | --- | --- |
|  | **B** | **SE** | **β** | **t** | **p** | **Lower** | **Upper** |
| (Intercept) | 4.300 | 0.284 |  | 15.161 | < .001 | 3.740 | 4.860 |
| age | 0.014 | 0.006 | 0.190 | 2.348 | 0.020 | 0.002 | 0.025 |
| sex_dummy | -0.205 | 0.137 | -0.119 | -1.499 | 0.136 | -0.475 | 0.065 |
| estrat | -0.053 | 0.055 | -0.080 | -0.970 | 0.334 | -0.161 | 0.055 |
| ideolo | -0.029 | 0.041 | -0.060 | -0.705 | 0.482 | -0.109 | 0.052 |
| religi | -0.012 | 0.066 | -0.015 | -0.185 | 0.854 | -0.143 | 0.118 |

***Coefficients Community Identification (Adj. R^2^ =.049)***

|  | | | | | | **95% CI** | |
| --- | --- | --- | --- | --- | --- | --- | --- |
|  | **B** | **SE** | **β** | **t** | **p** | **Lower** | **Upper** |
| (Intercept) | 4.116 | 0.261 |  | 15.760 | < .001 | 3.600 | 4.631 |
| age | 0.011 | 0.005 | 0.158 | 1.982 | 0.049 | 3.600×10^-5^ | 0.021 |
| sex_dummy | -0.412 | 0.126 | -0.257 | -3.274 | 0.001 | -0.660 | -0.163 |
| estrat | 0.011 | 0.050 | 0.017 | 0.209 | 0.835 | -0.089 | 0.110 |
| ideolo | 0.006 | 0.038 | 0.014 | 0.165 | 0.869 | -0.068 | 0.080 |
| religi | -0.008 | 0.061 | -0.011 | -0.131 | 0.896 | -0.128 | 0.112 |

***Coefficients National Identification (Adj. R^2^ = .019)***

|  | | | | | | **95% CI** | |
| --- | --- | --- | --- | --- | --- | --- | --- |
|  | **B** | **SE** | **β** | **t** | **p** | **Lower** | **Upper** |
| (Intercept) | 3.520 | 0.284 |  | 12.376 | < .001 | 2.958 | 4.082 |
| age | 0.010 | 0.006 | 0.141 | 1.739 | 0.084 | -0.001 | 0.022 |
| sex_dummy | -0.244 | 0.137 | -0.142 | -1.782 | 0.077 | -0.515 | 0.026 |
| estrat | 0.039 | 0.055 | 0.059 | 0.718 | 0.474 | -0.069 | 0.148 |
| ideolo | 0.026 | 0.041 | 0.054 | 0.630 | 0.529 | -0.055 | 0.106 |
| religi | 0.035 | 0.066 | 0.044 | 0.535 | 0.593 | -0.095 | 0.166 |

***Coefficients IWAH Bond (Adj. R^2^ =.017)***

|  | | | | | | **95% CI** | |
| --- | --- | --- | --- | --- | --- | --- | --- |
|  | **B** | **SE** | **β** | **t** | **p** | **Lower** | **Upper** |
| (Intercept) | 2.312 | 0.364 |  | 6.345 | < .001 | 1.592 | 3.032 |
| age | 0.001 | 0.008 | 0.015 | 0.180 | 0.858 | -0.014 | 0.016 |
| sex_dummy | 0.049 | 0.176 | 0.022 | 0.281 | 0.779 | -0.297 | 0.396 |
| estrat | 0.149 | 0.070 | 0.176 | 2.120 | 0.036 | 0.010 | 0.289 |
| ideolo | 0.019 | 0.052 | 0.031 | 0.365 | 0.716 | -0.084 | 0.123 |
| religi | 0.095 | 0.085 | 0.091 | 1.113 | 0.267 | -0.073 | 0.262 |

***Coefficients IWAH CONCERN (Adj. R^2^ = .00)***

|  | | | | | | **95% CI** | |
| --- | --- | --- | --- | --- | --- | --- | --- |
|  | **B** | **SE** | **β** | **t** | **p** | **Lower** | **Upper** |
| (Intercept) | 3.569 | 0.357 |  | 9.984 | < .001 | 2.863 | 4.275 |
| age | 0.008 | 0.007 | 0.089 | 1.089 | 0.278 | -0.007 | 0.023 |
| sex_dummy | -0.285 | 0.172 | -0.133 | -1.657 | 0.099 | -0.625 | 0.055 |
| estrat | 0.058 | 0.069 | 0.070 | 0.838 | 0.403 | -0.079 | 0.194 |
| ideolo | 0.027 | 0.051 | 0.044 | 0.518 | 0.605 | -0.075 | 0.128 |
| religi | -0.062 | 0.083 | -0.061 | -0.743 | 0.458 | -0.226 | 0.103 |
